# Targeted versus non-targeted HIV testing offered via electronic questionnaire in a Swiss emergency department: A randomized controlled study

**DOI:** 10.1371/journal.pone.0190767

**Published:** 2018-03-07

**Authors:** Cleo Gillet, Katharine E. A. Darling, Nicolas Senn, Matthias Cavassini, Olivier Hugli

**Affiliations:** 1 Faculty of Biology and Medicine, University of Lausanne, Lausanne, Switzerland; 2 Infectious Disease Service, Lausanne University Hospital, Lausanne, Switzerland; 3 Department of Ambulatory Care and Community Medicine, University of Lausanne, Lausanne, Switzerland; 4 Emergency Department, Lausanne University Hospital, Lausanne, Switzerland; TNO, NETHERLANDS

## Abstract

**Background:**

In Switzerland, the national HIV testing recommendations propose targeted testing. Although the emergency department (ED) is mentioned specifically as a site where HIV testing should take place, the testing rate in our ED is 1% of patients seen. The aim of this study was to use electronic tablets to offer testing to ED patients and to examine whether non-targeted screening increased testing rates compared to targeted testing.

**Methods:**

This randomised, cross-over design study took place at Lausanne University Hospital, Switzerland, between August and November 2015. Eligible patients were randomised to a targeted testing or a non-targeted screening arm. Using electronic tablets, targeted arm patients completed a risk factor assessment; patients with risk factors were offered free rapid HIV testing. Non-targeted arm patients received information about HIV and HIV testing on their tablet and were then offered testing. In a second step, patients who declined testing were crossed over to the other strategy. The primary endpoint was the HIV testing rate per arm.

**Results:**

Eighty patients were recruited to each study arm. In the targeted arm, 17 patients (of 80, 21%) had at least one risk factor and were offered testing, of whom eight (of 17, 47%) accepted. HIV testing rate in the targeted arm was 10% (8/80) compared to 48% (38/80) in the non-targeted arm (*P*<0.001). Secondary cross–screening, where targeted arm patients without risk factors were offered non-targeted screening, increased the testing rate in the targeted arm to 45% (36/80). Among patients offered testing, the acceptance rate did not differ between targeted and non-targeted arms, at 48% and 53%, respectively (*P* = 0.9)

**Discussion:**

In our centre, non-targeted HIV screening resulted in a higher testing rate than targeted testing due to more patients being offered a test. The acceptance rate of testing offered did not differ between targeted and non-targeted arms. Electronic tablets were well-received by patients and easy to use. We conclude that non-targeted HIV screening using electronic tablets would increase the HIV testing rate in our ED.

**Trial registration:**

ClinicalTrials.gov NCT03038724

## Introduction

In Switzerland, between 13,000 and 29,000 people are living with HIV [1–3], of whom an estimated 20% is unaware of being infected [3]. To address undiagnosed HIV infection, the Swiss Federal Office of Public Health (FOPH) has published HIV testing recommendations since 2007 which propose targeted testing [4]. With this approach, individuals are offered testing if they present symptoms or signs listed in the FOPH recommendations as being HIV testing indications, or if they belong to a group at high risk of HIV acquisition, defined as men who have sex with men (MSM), intravenous drug users (IVDUs), persons from countries of high HIV prevalence, notably sub-Saharan Africa (SSA), or sexual partners of people in these groups [5, 6].

In 2010, the Emergency Department (ED) was mentioned specifically by the FOPH as a site where HIV testing should occur [5]. This is justified by the high number of patients using this service (in Switzerland in 2011, this was 1.6 million visits or 20.4 per 100 inhabitants [7]) and by ED patient demography, many individuals being vulnerable and at risk of HIV acquisition. For individuals with poor access to health care, and for healthy individuals who consult infrequently, a visit to the ED may represent the only opportunity for HIV testing [8]. However, we reported that the 2010 FOPH recommendations made no difference to testing practice and that only 1% of ED patients seen were tested for HIV [9]. In our centre, where local HIV seroprevalence is 0.2–0.5% [1, 3], we have reported that few ED doctors are aware of the national testing recommendations, and that being aware is not associated with performing more tests [10]. Furthermore, ED doctors detected only 30% of patients who had indications for HIV screening and failed to offer HIV testing even when indications were identified [11].

Whilst the FOPH recommends targeted testing in Switzerland, no randomized study has been performed to determine whether this is the best approach in Swiss EDs. An alternative to targeted testing is non-targeted screening, where all individuals are offered testing when seen by a medical practitioner, whatever their reason for presenting. Non-targeted screening may be easier to implement as this approach does not rely on busy ED doctors who may be unskilled at recognising risk factors or who find initiating such a discussion awkward [12]. In the United States (US), the Centers for Disease Control and Prevention (CDC) recommended non-targeted screening a decade ago [13]. More recently, in Europe, France has adopted the same approach, in areas where HIV seroprevalence is >0.1% [14], as has the United Kingdom, in areas where seroprevalence is >0.2% [15]. We have reported testing rates of 50% among ED patients offered non-targeted testing by research assistants working in parallel to the patient-doctor consultation [11]. However, while we have observed patient acceptance of testing in the ED to be high [11, 16], employing additional staff to screen for HIV is costly.

One method of delivering information, offering HIV testing and preventive services to patients without increasing staff workload is by using electronic tablets [17]. Tablets have been shown to be easy to use [18] and, in the context of HIV, risk behaviour is more willingly disclosed to an electronic intermediary than to a health care provider [19, 20]. An electronic survey could adapt itself to patient answers, offering a dynamic and interactive experience [21] and leading to longer-term effects on patient risk behaviour perception [22]. In the ED, patients could use such tablets, and engage with their health care, while waiting for treatment.

In this study, we used electronic tablets to offer HIV testing to ED patients and to examine whether targeted testing or non-targeted screening would result in higher testing rates.

## Methods

### Ethics statement

This study was approved by the ethics committee on human research of the Canton of Vaud, Switzerland (protocol N°228/15; approved 27^th^ July 2015)**.** All participants signed written informed consent and all data were anonymised for analysis.

### Setting and participants

The study took place in the ED of Lausanne University Hospital (LUH) between 11^th^ August and 28^th^ November 2015. LUH is a 1,500-bed teaching hospital which serves as a primary-level community hospital for Lausanne (catchment population 300,000) and as a secondary and tertiary referral hospital for sectors of Western Switzerland (catchment population 1–1.5 million), receiving around 40,000 patient visits annually [7].

In this study, all clinically stable patients aged 18 to 75 years presenting to LUH ED were eligible. Exclusion criteria were: admission >12 hours prior to eligibility screening; transfer from another hospital or prison; known positive HIV status or HIV testing offered by the ED physician before the study investigator approached the patient; clinical instability and inability to provide informed consent.

The study investigator, a medical student who was not involved in patient care, screened all admitted patients for eligibility using the live ED patient flow software during 30 eight-hour shifts. Patients were invited to take part in a study about screening. To limit response bias, the exact nature of the study was not presented until the end of the questionnaire. For patients declining to participate, demographic data were recorded.

### Study design and questionnaires

The study was randomised with two arms: targeted testing and non-targeted screening (S1 Text. Original Study Protocol). The trial was registered on the ClinicalTrials website after the enrolment of patients was complete (ClinicalTrials.gov ID NCT03038724) (S1 Text. Clinical Trials Protocol). The reason for not registering this trial prior to enrolment was that it began as a medical student’s Master’s project, and such registration was not required by the medical faculty. The authors confirm that the trial was registered at ClinicalTrials as originally designed and that no ongoing or related trials for this intervention are underway that would require registration.

The primary objective was to determine whether non-targeted screening was superior to targeted testing using FOPH testing criteria [6] in terms of the number of free rapid HIV tests performed among eligible patients during their ED visit.

Patients consenting to participate were randomised to one of the two arms in a 1:1 proportion based on a sequence determined by random blocks of four and six generated by the website www.randomisation.com. Allocations were prepared by an independent researcher (OH), and placed inside sealed opaque envelopes numbered consecutively. Envelopes were opened by the study investigator in sequence and the date of opening was written on the envelope. Sequential envelope opening was strictly audited by the lead investigator (OH). Once randomized, patients were presented with an electronic tablet displaying the questionnaire appropriate to the assigned arm, using the website SurveyMonkey® (S3 Text. Study Questionnaire). While patients completed the questionnaire, the study investigator remained outside the examination cubicle but was available to assist with questions.

The targeted arm questionnaire asked patients about their HIV risk factors based on FOPH HIV testing criteria [6], requesting participants to state whether the following was applicable: being a MSM or IVDU, having USI with MSM, IVDUs, sex worker(s) or partner(s) with a sexually-transmitted infection (STI) or known to be HIV positive, USI with partners from SSA, the Caribbean, Eastern Europe or South-East Asia or while travelling in these regions. If the patient responses revealed at least one HIV risk factor, the tablet informed the patient that HIV testing was recommended and offered rapid HIV testing, using the following script:

‘Thank you for your answers on HIV risk behaviour. According to your answers, you have risk factors for HIV infection. According to official recommendations, you should be screened for HIV. The investigator can test you right now with a rapid test. It is free of charge and the result will be available in 3 to 6 minutes. Do you wish to get tested for HIV with a rapid HIV test?’

An additional question on risk behaviour in the targeted arm asked patients if they had engaged in unprotected sexual intercourse (USI) since their last negative HIV test. This scenario is not mentioned specifically in the FOPH recommendations but is mentioned by the US Preventive Services Task Force Recommendation Statement on HIV screening [1, 23]. The question was included to examine whether it increased the HIV testing rate over that resulting from questions based solely on the FOPH testing recommendations.

In the non-targeted arm, the patients were provided with information about HIV and the pros and cons of rapid HIV testing. They were then offered rapid testing without undertaking a risk factor assessment as follows:

‘Having read this information, would you like to get tested for HIV with a rapid test right now?’

### Cross-screening

The secondary study objective was to examine the effect of a cross-screening strategy on the HIV testing rate. Patients randomized to the targeted arm who reported no risk factors were automatically offered non-targeted screening as follows:

‘According to the answers you have given, you do not have any risk factors for HIV infection. Do you wish to take the opportunity to get tested anyway? The test uses a drop of blood taken from the tip of the finger and gives a result in 3 to 6 minutes. It is free of charge’.

In the case of patient responses not allowing a risk assessment to be made, non-targeted screening was also offered as follows:

‘The answers you have given in this questionnaire do not allow us to evaluate whether or not you are at risk of HIV infection. Do you wish to take the opportunity to get tested anyway? The investigator could test you right now and it is free of charge’.

The cross-screening strategy was also applied to patients randomized to the non-targeted arm who declined non-targeted HIV screening. These patients were automatically directed by the tablet to the targeted testing questionnaire, with an HIV risk factor assessment and the offer of rapid testing if risk factors were present. In both cross-screening arms, patients were asked to give reasons if they opted in the end to decline testing.

For each completed questionnaire, the tablet displayed only the patient’s decision to accept or decline testing; other questionnaire responses were hidden from the study investigator. Additional data were gathered using a paper questionnaire covering patient socio-economic status, ease of tablet use and attitudes towards the ED as a site for HIV screening. The evaluation of patient attitudes to ED HIV screening consisted of two statements: 1) ‘The emergency department is a suitable place to offer HIV screening’ and 2) ‘I am in favour of routine HIV screening tests to be offered to all patients in the ED’. Patients rated these statements using a 5-level Likert scale (From “strongly agree” to “strongly disagree”). For analysis, the 2 categories “strongly agree” and “agree” were pooled.

Patients accepting rapid testing signed a second written informed consent which included information about the study and about the INSTI rapid HIV test (24 INSTI™ HIV-1/HIV-2 Rapid Antibody Test, BioLytical Laboratories, Richmond, BC, Canada). Rapid testing was conducted by the study investigator using finger prick blood. A negative test was announced immediately to the patient by the study investigator. In the event of a reactive test, the result would be announced by an infectious diseases specialist who would coordinate confirmatory testing and linkage to care. To ensure continuous specialist availability, consecutive eligible patients were approached during eight-hour shifts between 08:00 and 20:00 when the investigator was present, representing therefore a convenience sample. To minimize sampling bias, the shifts took place seven days a week.

### Statistical analysis

The sample size was determined prior to the initiation of the study. Assuming an increase in the testing rate from 11% in the targeted arm to 30% in the non-targeted arm, a sample of 80 patients per arm would provide a power of 80%, with a two-sided alpha of 0.05. The figure of 11% was based on the published observation that an audio computer self interview–based feedback on reported HIV risk behaviors increased patient awareness of being at risk by 11%, generating an opportunity for HIV screening [24]. At the time the current study protocol was developed, an ongoing investigation at our centre demonstrated that around 30% of patients accepted rapid HIV-testing in the ED setting (although the final published figure was 34%) [16].

Patients were analysed according to demographic parameters where education level was categorised according to the United Nations International Standard Classification of Education (ISCED) and profession level according to the International Labour Organisation International Standard Classification of Occupations (ISCO-08).

Data are presented as mean ± standard deviation (SD), median and inter-quartile range (IQR) or as percentages. Proportions were compared using the Chi-squared test or Fisher’s exact test as appropriate. Means were compared using Student’s t-test. For the primary outcome, proportions with 95% confidence intervals with Wilson’s confidence intervals were estimated. Statistical analyses were conducted using Stata version 14 (StataCorp, College Station, TX, USA).

## Results

### Participants

**Fig 1:**
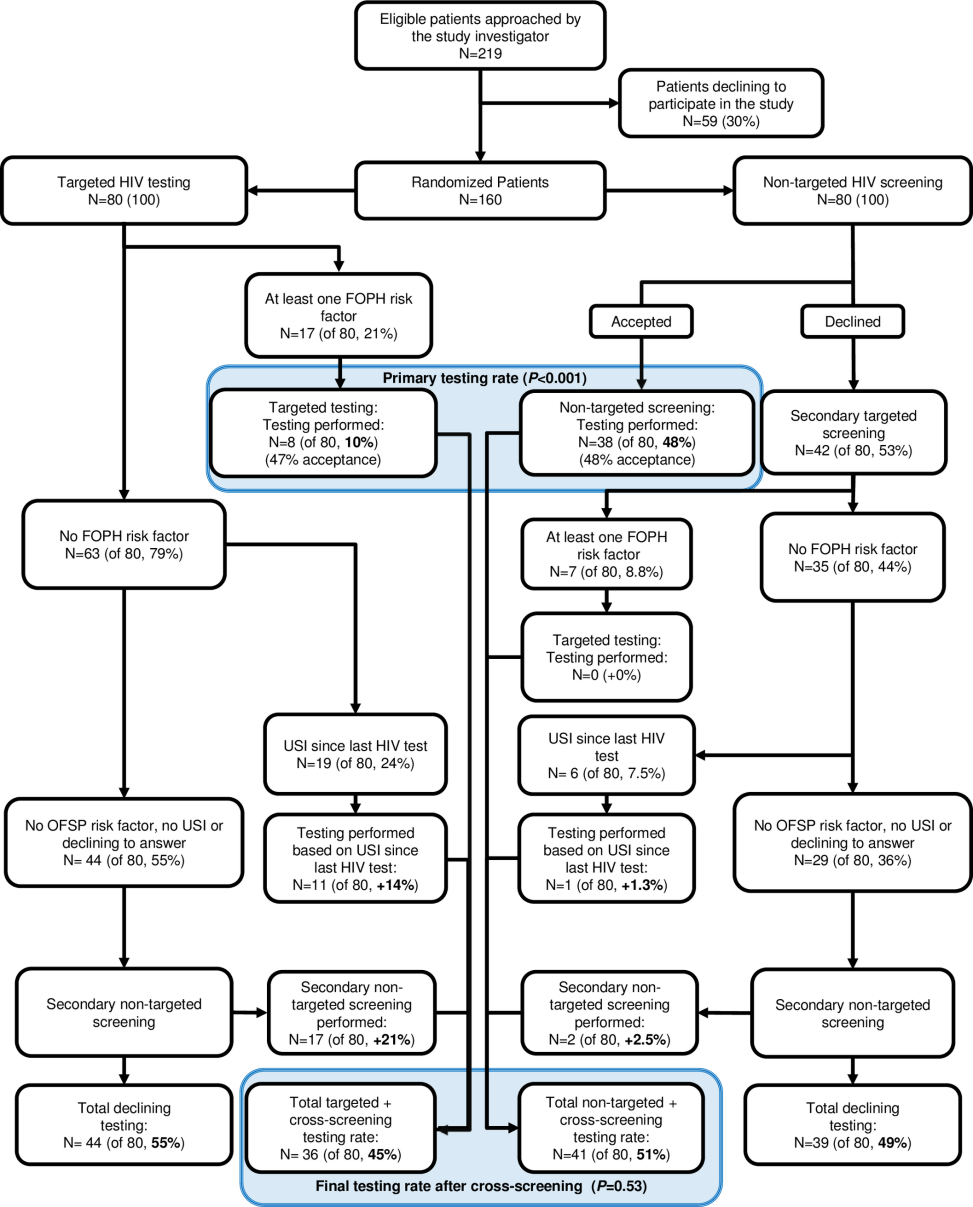
Flowchart diagram demonstrating primary and final HIV testing rates with targeted testing and non-targeted screening following randomisation of 160 patients presenting to the emergency department. Abbreviations: FOPH, Federal Office of Public Health; USI, unprotected sexual intercourse.

Of 219 patients screened and found to be eligible, 30% declined to participate prior to randomisation (Fig 1).

There was no difference in age or gender between those who declined and those who participated in the study. In all, 160 consenting patients were randomised, 80 to each arm, among whom the mean age was 42.7 years. The demographic characteristics of participating patients are shown in Table 1.

**Table 1:** Demographic characteristics of participating patients by study arm. Abbreviations: ED, Emergency department; SD, Standard deviation.

	Targeted arm (N = 80)	Non-targeted arm (N = 80)
**Mean age** (SD)	41.8 (15.3)	43.6 (16.5)
**Female gender,** n (%)	42 (53%)	32 (40%)
**Nationality**, n (%)		
Swiss	53 (66%)	54 (67%)
Non-Swiss European	22 (28%)	23 (29%)
Other	5 (6%)	3 (4%)
**Marital status**, n (%)		
Married	31(39%)	31 (39%)
Single	32 (40%)	35 (44%)
Divorced	15 (19%)	12 (15%)
Widowed	2 (2%)	2 (2%)
**Highest Education level**, n (%)^1^		
Tertiary education	30 (38%)	27 (34%)
Post-secondary non-tertiary / Upper secondary / Lower secondary education	39 (49%)	45 (56%)
Primary/ Pre-primary education	11 (14%)	8 (10%)
**Occupation status**, n (%)		
Employed	51 (64%)	46 (58%)
Seeking employment/ home maker/ Student	19 (24%)	16 (20%)
Retired	10 (13%)	17 (21%)
**Occupation category**, n (%)^2^		
Manager / Professional	28 (35%)	26 (33%)
Technical / clerical worker	29 (36%)	25 (31%)
Skilled manual worker	10 (13%)	21 (26%)
Unskilled occupation	13 (16%)	8 (10%)
**Have a primary care physician**, n (%)	72 (90%)	68 (85%)
**Presenting with trauma**, n (%)	18 (23%)	30 (38%)
**ED Minor section**, n (%)	61 (76%)	63 (79%)
**Discharge status**, n (%)		
Discharged	61 (76%)	71 (89%)
Admitted	16 (20%)	9 (11%)
Unknown	1 (1%)	4 (5%)

^1^ Based on the International Standard Classification of Education (ISCED)

^2^ Based on the International Standard Classification of Occupations (ISCO-08)

### Primary results

In the targeted arm, 17 patients (of 80, 21%) reported HIV risk factors and eight patients accepted testing (of 80, 10%, Fig 1). The question on USI since the last negative HIV test resulted in an additional 11 patients accepting testing (+14% of the targeted arm). In the non-targeted screening arm, 38 patients accepted the offer of testing, giving a testing rate of 48% (38/80).

### Secondary results

The secondary study objective was to examine the yield of cross-screening strategies. Directing patients initially assigned to the targeted arm and who did not report risk factors to the non-targeted arm resulted in 17 more patients accepting testing (+21%) (Fig 1). The overall testing rate was therefore 36/80 (45%) in the targeted arm (Fig 1).

In the non-targeted arm, seven patients reported HIV risk factors and six reported USI, of whom one accepted testing (+1.3%, Fig 1). The total combined testing rate after cross-screening was 41/80 (51%) for the non-targeted arm (Fig 1). In this way, testing was performed in 17/44 patients (39%) offered a secondary non-targeted approach and by 3/42 patients (7.1%) of patients offered a secondary targeted approach. No rapid HIV test performed was reactive.

Patient acceptance rate of testing offered was not significantly different between the two arms, at 48% for the non-targeted arm (38/80) and 53% for the targeted arm (19/36) (*P* = 0.9). However, because only patients with risk factors were offered testing in the targeted arm, the testing rate in this arm was lower than that in the non-targeted arm at 10% (8/80) compared to 48% (38/80) (*P*<0.001, Fig 1), or 24% (19/80) compared to 48% (38/80) (*P* = 0.003) if including patients offered testing in the targeted arm through reporting USI since their last HIV test.

### HIV risk factor questionnaire

The most common risk factors reported in the targeted arm were USI since the last negative HIV test, USI with people from high-prevalence areas or whilst in a high HIV-prevalence area, or with partners with a STI (Table 2).

**Table 2:** HIV risk factors among targeted-arm patients accepting or declining the offer of rapid HIV testing.

	Accepting test n (%) N = 36	Declining test n (%) N = 44
USI since last HIV test	11 (31%)	8 (18%)
USI with person(s) of high-prevalence areas or while travelling through such areas^1^	4 (11%)	4 (9.1%)
USI with partner(s) with STI	4 (11%)	2 (4.6%)
MSM	1 (2.8%)	2 (4.6%)
USI with sex worker(s)	0	3 (6.8%)
USI with partner(s) known to be HIV positive	0	1 (2.3%)
IVDU or USI with IVDU	0	1 (2.3%)

^1^Seven patients in this group had an additional risk factor

Abbreviations: USI, unprotected sexual intercourse; STI, sexually-transmitted infection; MSM, men who have sex with men; IVDU, intravenous drug user.

Of all patients who answered the HIV risk factor questionnaire, 60% reported no risk factors (44 of 80 targeted-arm patients and 29 of 42 non-targeted arm patients, Fig 1). The reporting of risk factors was not more associated with accepting HIV testing in the targeted arm, and there was no association between the number of risk factors reported and testing rate (*P* = 0.95, Table 3). Reported risk factors did not differ significantly with age (mean age 46±15 versus 41±15; *P* = 0.17) or gender (female gender: 59% versus 51%; *P* = 0.60) for those with and without HIV risk factors, respectively.

**Table 3:** Association between the number of Federal Office of Public Health (FOPH) HIV risk factors and HIV rapid test acceptance among patients assigned to the targeted arm.

	Accepting test, n (%)^1^ N = 36	Declining test, n (%)^1^ N = 44	*P*
**Number of HIV risk factors (RFs)**			0.95
No RFs	17 (47)	27 (61)	
USI since last HIV test as only RF	11 (31)	8 (18)	
1 FOPH RF	6 (17)	6 (14)	
2 FOPH RFs	2 (5.6)	2 (4.6)	
3 FOPH RFs	0 (0)	1 (2.3)	

^1^Percentages may not add up to 100 due to rounding.

### Patients declining testing

In total, 83 patients (54%) declined rapid testing by the end of the initial arm assignment and cross-screening, 44 patients (55%) initially assigned to targeted testing and 39 patients (49%) initially assigned to non-targeted screening (Fig 1 and Table 4). Among the 83 patients declining testing, 29 (35%) reported risk factors, 24 (29%) had never previously been tested for HIV, and 16 (19%) had been tested more than five years ago. The two main reasons for declining testing were a recent negative test and the self-perception of not being at risk (Table 4). There was no difference by age or gender between participants declining testing in either arm.

**Table 4:** Questionnaire responses from participants declining HIV testing.

	Targeted arm N = 44	Non-targeted arm N = 39	*P*-Value
**Reported Risk Factors**			
USI without other risk factor	8 (18%)	5 (13%)	0.77
FOPH risk factors +/- USI	9 (20%)	7 (18%)	
**Previous HIV test**	31 (70%)	28 (72%)	1
**Main reason for declining testing**			
Do not consider self at risk for HIV	20 (45%)	13 (33%)	0.01
Recent negative HIV test	6 (14%)	16 (41%)	
Prefer to be tested by primary care physician	2 (5%)	2 (5%)	
Fear of test result	1 (2%)	3 (8%)	
Fear of family/employer seeing the result	1 (2%)	1 (3%)	
No time	0	1 (3%)	
Other reason	12 (27%)	2 (5%)	
Do not wish to answer	2 (5%)	1 (3%)	

Abbreviations: USI, Unprotected Sexual Intercourse

### Patient attitudes to HIV screening in the ED

Of all study patients, 140 (of 160, 88%) were in favour of using waiting periods in the ED to screen for chronic diseases such as diabetes. A similar number (143 of 160, 89%) considered the ED an appropriate place to offer HIV screening and 136 patients (of 160, 85%) were in favour of non-targeted HIV screening in the ED. Considering the ED as an unsuitable place for screening was associated with higher rates of declining testing (*P* = 0.044).

### Use of electronic tablets

Nine participants (of 160, 5.6%) required technical help from the study investigator to use the tablet, and six (3.8%) required help to understand the questionnaire. In total, 157 participants (of 160, 98%) declared the tablet easy to use, including those who initially required assistance.

## Discussion

In our ED setting, where national HIV testing recommendations propose targeted testing, the number of HIV tests performed was significantly higher with non-targeted screening than with targeted testing through more patients being offered testing. Patient acceptance of testing was almost 50% and was not significantly different between the two testing approaches. Cross-screening—offering non-targeted screening to patients assigned to the targeted arm who had no HIV risk factors—increased the final testing rate from 8/80 (10%) to 36/80 (45%). Cross-screening the other way—directing participants declining non-targeted screening to the targeted testing questionnaire—had a marginal effect on the final testing rate. Finally, electronic tablets were feasible for ED patients to use, regardless of age, and most participants considered the ED an appropriate setting for HIV screening.

This is the first randomised, prospective study to examine HIV testing approaches in the ED setting in Switzerland, a country with universal health coverage. Other studies have compared targeted versus non-targeted testing in US EDs, using healthcare professionals rather than electronic tablets. Lyons *et al* randomised patients to targeted or non-targeted testing in an urban ED and reported that non-targeted testing resulted in more HIV tests being performed and consequently more new HIV diagnoses than with targeted testing [25]. Although targeted testing had a lower proportion of ED patient encounters compared with non-targeted screening, patient acceptance was significantly higher at 47% compared to 41% (*P*<0.002). Haukoos *et al*, again in an urban ED centre, compared a four-month phase of non-targeted screening to a four-month phase of ‘enhanced’ targeted testing using the Denver HIV Risk Score to identify patients with increased probability of undiagnosed HIV [26]. In this study, fewer tests were required to identify the same number of new HIV infections during the enhanced targeted testing phase, and patient acceptance of testing was higher, compared to non-targeted screening phase. Both these US studies examined diagnostic yield of testing as their primary endpoint.

A third US study examined the effect of testing approach, placing patient acceptance as the primary endpoint [27]. In this study by Montoy *et al*, ED staff informed patients that rapid testing was being offered to all patients and then offered testing in one of three ways: ‘You can let me, your nurse, or your doctor know if you'd like a test today’ (opt-in); ‘Would you like a test today?’ (active choice); or ‘You will be tested unless you decline’ (opt-out). The authors reported that active-choice and opt-out screening significantly increased testing acceptance among all patient risk groups compared to opt-in testing [27]. The active choice approach most resembles the way in which testing was offered to our patients. Our study and that of Montoy *et al* highlights the importance of not only deciding whom to screen but how to offer the test.

We chose to add to the FOPH list of risk factors a risk assessment question about USI following a previous negative HIV test, as recommended by the US Preventive Task Force in 2013 [23]. This risk factor was reported frequently among study participants and led to more tests being performed than when testing was based on FOPH risk factors alone. This observation has implications for improving targeted screening in Switzerland.

The main reason for declining testing among targeted arm patients was the perception of not being at risk, even when risk factors were reported, the most frequent being USI since the last negative test. We also observed that reporting risk factors was not significantly positively-associated with testing acceptance. It is possible that the ‘at-risk’ label given by the tablet to targeted arm patients negatively influenced acceptance. Indeed, it has been observed that, even in a confidential setting, patients’ fear of stigmatization and potential lack of confidentiality may negatively influence testing acceptance when sensitive data might be collected [28]. In addition, self-perceived risk may differ from real risk [24, 29] for reasons of denial, distancing or downward comparison [30]. Patient evaluation of HIV risk may depend on their type of risk factor and their level of education [24, 31]. One means of addressing inaccurate risk perception would be to add an interactive information module to the tablet, where at-risk patient behaviours could be categorised and presented back to the patient in real-time. This aspect will be explored further using tablets in our centre. However, it is already encouraging at this stage that the patient acceptance rate of testing offered via tablet in this study (47–48%) was only slightly lower than that observed at our centre when testing was offered using the same language but via a health care practitioner (50%) [11].

This study has limitations. As no tests performed were positive, our study does not provide insight into which strategy is diagnostically most effective, only which strategy leads to more HIV tests being performed. The higher number of tests performed in the non-targeted arm would not necessarily lead to a higher number of new HIV diagnoses being made, compared to the targeted approach. This limitation is related to the chosen primary outcome of the study, made prior to the study being conducted, and is not related to the absence of positive tests; given the HIV seroprevalence in our catchment population, we would need to conduct many more than 80 tests per study arm to make one new HIV diagnosis. Data from previous studies in the US ED setting comparing targeted and non-targeted (or ‘universal’) approaches give mixed results as described above [32][25]. Our study was monocentric with a convenience sample of ED patients; patients were approached between 08:00 and 20:00 to enable specialist referral in the event of a reactive rapid test. Against this limitation, patients admitted within the preceding 12 hours were eligible and consecutive patients were recruited every day of the week. However, not all patients presenting to the ED could be surveyed, a source of potential selection bias which would limit the generalizability of our findings. We did not assess risk factors among patients in the non-targeted arm who accepted testing. We cannot therefore determine whether, despite randomisation, patients in the non-targeted arm were more at risk of HIV acquisition. However, demographic profiles were similar between the two arms and the non-targeted approach was acceptable to targeted arm patients who initially declined testing. Equally, patients in the targeted arm received less detailed information on HIV and the advantages of testing than those in the non-targeted arm and this may have introduced bias. Finally, although we demonstrated feasibility of HIV screening using electronic tablets in the ED, regardless of the study arm to which patients were assigned, we did not examine which testing approach would be easier to implement in terms of the time and resources required. However, in principle, the time required is the time during which the patient is waiting to be seen and the resources required, beyond the tablets themselves, would depend on whether salaried ED staff or supplementary staff carry out testing and whether this would take place only when a duty HIV specialist is present, or 24 hours a day.

In conclusion, non-targeted HIV screening resulted in higher HIV testing rates than targeted testing in a country where targeted testing is currently recommended. Testing in the ED using electronic tablets was well-received by patients and provided a potential medium for offering preventive services without straining the medical team. Our results provide evidence that implementing non-targeted HIV screening would increase the number of HIV tests performed in our ED.

## Supporting information

S1 Checklist CONSORT_Checklist. (DOC)

S1 Text Original_study_protocol. (DOC)

S2 Text ClinicalTrials_Study_protocol. (DOC)

S3 Text Study_questionnaire. (DOC)

S1 Fig CONSORT_Flow_Diagram. (TIF)

## References

[1] UNAIDS epidemiology figures 2013: Switzerland http://www.unaids.org/sites/default/files/epidocuments/CHE.pdf2013 [

[2] Office fédéral de la santé publique: VIH et IST en 2013: tendances du premier semestre. Bulletin de l'OFSP. 2013;47:847–50.

[3] Kohler P, Schmidt AJ, Cavassini M, Furrer H, Calmy A, Battegay M, et al The HIV care cascade in Switzerland: reaching the UNAIDS/WHO targets for patients diagnosed with HIV. AIDS. 2015;29(18):2509–15. doi: 10.1097/QAD.0000000000000878 26372488 10.1097/QAD.0000000000000878

[4] Office fédéral de la santé publique. Dépistage du VIH et conseil initiés par les médecins. https://www.bag.admin.ch/bag/fr/home.html. Bulletin de l'OFSP. 2007;21:371–3.

[5] Office fédéral de la santé publique. Dépistage du VIH effectué sur l’initiative des médecins: recommandations pour les patients adultes. https://www.bag.admin.ch/bag/fr/home.html Bulletin de l'OFSP. 2010(11/10):364–6.

[6] Office fédéral de la santé publique. Dépistage du VIH effectué sur l’initiative des médecins en présence de certaines pathologies (maladies évocatrices d’une infection à VIH) https://www.bag.admin.ch/bag/fr/home.html. Bulletin de l'OFSP 2013:1–5.

[7] Vilpert S. Consultations dans un service d'urgence en Suisse. Neuchâtel: Observatoire suisse de la santé, 2013 Report No. 3/2013. 2013.

[8] Darling KE, Gloor E, Ansermet-Pagot A, Vaucher P, Durieux-Paillard S, Bodenmann P, et al Suboptimal access to primary healthcare among street-based sex workers in southwest Switzerland. Postgrad Med J. 2012. 10.1136/postgradmedj-2012-131001 23150609

[9] Darling KE, Hugli O, Mamin R, Cellerai C, Martenet S, Berney A, et al HIV Testing Practices by Clinical Service before and after Revised Testing Guidelines in a Swiss University Hospital. PLoS One. 2012;7(6):e39299 doi: 10.1371/journal.pone.0039299 22761757 10.1371/journal.pone.0039299 PMC3386253

[10] Darling KE, de Allegri N, Fishman D, Kehtari R, Rutschmann OT, Cavassini M, et al Awareness of HIV testing guidelines is low among Swiss emergency doctors: a survey of five teaching hospitals in French-speaking Switzerland. PLoS One. 2013;8(9):e72812 doi: 10.1371/journal.pone.0072812 24039804 10.1371/journal.pone.0072812 PMC3765151

[11] De Rossi N, Dattner N, Cavassini M, Peters S, Hugli O, Darling KEA. Patient and doctor perspectives on HIV screening in the emergency department: A prospective cross-sectional study. PloS one. 2017;12(7):e0180389 doi: 10.1371/journal.pone.0180389 28732088 10.1371/journal.pone.0180389 PMC5521743

[12] Epstein RM, Morse DS, Frankel RM, Frarey L, Anderson K, Beckman HB. Awkward moments in patient-physician communication about HIV risk. Ann Intern Med. 1998;128(6):435–42. 9499326 10.7326/0003-4819-128-6-199803150-00003

[13] Branson BM, Handsfield HH, Lampe MA, Janssen RS, Taylor AW, Lyss SB, et al Revised recommendations for HIV testing of adults, adolescents, and pregnant women in health-care settings. Morbidity & Mortality Weekly Report Recommendations & Reports. 2006;55(RR-14):1–17; quiz CE1-4. 16988643

[14] Haute autorité de santé. HIV infection screening in France. Screening Strategies. Executive summary and guidelines. 2009 [Available from: https://www.has-sante.fr/portail/upload/docs/application/pdf/2010-02/hiv_infection_screening_in_france_-_screening_strategies_-_executive_summary_2010-02-26_10-28-32_643.pdf (Accessed 10 Aug 2017).

[15] Pradeep TG, Gangasagara SB, Subbaramaiah GB, Suresh MB, Gangashettappa N, Durgappa R. Prevalence of undiagnosed HIV infection in patients with ocular surface squamous neoplasia in a tertiary center in Karnataka, South India. Cornea. 2012;31(11):1282–4. doi: 10.1097/ICO.0b013e3182479aed 22673850 10.1097/ICO.0b013e3182479aed

[16] Favre-Bulle T, Baudat D, Darling K, Mamin R, Peters S, Cavassini M, et al Patients' understanding of blood tests and attitudes to HIV screening in the emergency department of a Swiss teaching hospital: a cross-sectional observational study. Swiss Med Wkly. 2015;145:w14206 doi: 10.4414/smw.2015.14206 26636370 10.4414/smw.2015.14206

[17] Burns F, Edwards SG, Woods J, Haidari G, Calderon Y, Leider J, et al Acceptability, feasibility and costs of universal offer of rapid point of care testing for HIV in an acute admissions unit: results of the RAPID project. HIV Med. 2013;14 Suppl 3:10–4. 10.1111/hiv.12056 24033896

[18] Choo EK, Ranney ML, Aggarwal N, Boudreaux ED. A systematic review of emergency department technology-based behavioral health interventions. Acad Emerg Med. 2012;19(3):318–28. doi: 10.1111/j.1553-2712.2012.01299.x 22435865 10.1111/j.1553-2712.2012.01299.x PMC5693241

[19] Adebajo S, Obianwu O, Eluwa G, Vu L, Oginni A, Tun W, et al Comparison of audio computer assisted self-interview and face-to-face interview methods in eliciting HIV-related risks among men who have sex with men and men who inject drugs in Nigeria. PLoS One. 2014;9(1):e81981 doi: 10.1371/journal.pone.0081981 24416134 10.1371/journal.pone.0081981 PMC3885382

[20] Jones J, Stephenson R, Smith DK, Toledo L, La Pointe A, Taussig J, et al Acceptability and willingness among men who have sex with men (MSM) to use a tablet-based HIV risk assessment in a clinical setting. Springerplus. 2014;3:708 doi: 10.1186/2193-1801-3-708 25525569 10.1186/2193-1801-3-708 PMC4265639

[21] Kreuter MW, Strecher VJ, Glassman B. One size does not fit all: the case for tailoring print materials. Ann Behav Med. 1999;21(4):276–83. 10721433 10.1007/BF02895958

[22] Portnoy DB, Scott-Sheldon LA, Johnson BT, Carey MP. Computer-delivered interventions for health promotion and behavioral risk reduction: a meta-analysis of 75 randomized controlled trials, 1988–2007. Prev Med. 2008;47(1):3–16. doi: 10.1016/j.ypmed.2008.02.014 18403003 10.1016/j.ypmed.2008.02.014 PMC2572996

[23] Moyer V. U. S. Preventive Services Task Force. Screening for HIV: U.S. Preventive Services Task Force Recommendation Statement. Ann Intern Med. 2013;159(1):51–60. doi: 10.7326/0003-4819-159-1-201307020-00645 23698354 10.7326/0003-4819-159-1-201307020-00645

[24] Merchant RC, Clark MA, Langan TJt, Seage GR 3rd, Mayer KH, DeGruttola VG. Effectiveness of increasing emergency department patients' self-perceived risk for being human immunodeficiency virus (HIV) infected through audio computer self-interview-based feedback about reported HIV risk behaviors. Acad Emerg Med. 2009;16(11):1143–55. doi: 10.1111/j.1553-2712.2009.00537.x 20053235 10.1111/j.1553-2712.2009.00537.x PMC3173950

[25] Lyons MS, Lindsell CJ, Ruffner AH, Wayne DB, Hart KW, Sperling MI, et al Randomized comparison of universal and targeted HIV screening in the emergency department. J Acquir Immune Defic Syndr. 2013;64(3):315–23. doi: 10.1097/QAI.0b013e3182a21611 23846569 10.1097/QAI.0b013e3182a21611 PMC4241750

[26] Haukoos JS, Hopkins E, Conroy AA, Silverman M, Byyny RL, Eisert S, et al Routine opt-out rapid HIV screening and detection of HIV infection in emergency department patients. JAMA. 2010;304(3):284–92. doi: 10.1001/jama.2010.953 20639562 10.1001/jama.2010.953

[27] Montoy JC, Dow WH, Kaplan BC. Patient choice in opt-in, active choice, and opt-out HIV screening: randomized clinical trial. BMJ. 2016;532:h6895 doi: 10.1136/bmj.h6895 26786744 10.1136/bmj.h6895 PMC4718971

[28] Chesney M, Smith A. Critical delays in HIV testing and care. The potential role of stigma Am Behav Sci. 1999;42(7):1162–74.

[29] Pisculli ML, Reichmann WM, Losina E, Donnell-Fink LA, Arbelaez C, Katz JN, et al Factors associated with refusal of rapid HIV testing in an emergency department. AIDS Behav. 2011;15(4):734–42. doi: 10.1007/s10461-010-9837-2 20978834 10.1007/s10461-010-9837-2 PMC3082047

[30] Gerrard M, Gibbons FX, Bushman BJ. Relation between perceived vulnerability to HIV and precautionary sexual behavior. Psychol Bull. 1996;119(3):390–409. 8668745 10.1037/0033-2909.119.3.390

[31] Takahashi TA, Johnson KM, Bradley KA. A population-based study of HIV testing practices and perceptions in 4 U.S. states. J Gen Intern Med. 2005;20(7):618–22. doi: 10.1111/j.1525-1497.2005.0112.x 16050856 10.1111/j.1525-1497.2005.0112.x PMC1490154

[32] Haukoos JS, Hopkins E, Byyny RL, Denver Emergency Department HIVTSG. Patient acceptance of rapid HIV testing practices in an urban emergency department: assessment of the 2006 CDC recommendations for HIV screening in health care settings. Ann Emerg Med. 2008;51(3):303–9, 9 e1. doi: 10.1016/j.annemergmed.2007.10.028 18191295 10.1016/j.annemergmed.2007.10.028

