# Supplementary material for: Targeted versus non-targeted HIV testing offered via electronic questionnaire in a Swiss emergency department: A randomized controlled study
Source: PLoS One. 2018 Mar 7;13(3):e0190767. doi: 10.1371/journal.pone.0190767 (PMC5841645; doi:10.1371/journal.pone.0190767)
Supplement: S1 Text — (DOC) [file pone.0190767.s002.doc]

# [
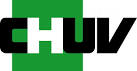
](http://www.google.ch/url?q=http://www.cliniquedudos.ch/fr/cv.html&sa=U&ei=BmAwU_z2Duiy7Ab2q4CoBg&ved=0CC8Q9QEwAQ&usg=AFQjCNHJiHnIRTgaIj_KqBXVvvkc3FrczA) [
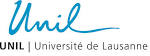
](http://www.google.ch/url?q=http://ceat.epfl.ch/&sa=U&ei=XWAwU-H8GY6rhAforoHQCA&ved=0CC8Q9QEwAQ&usg=AFQjCNE7XSjB3ccF0LlRcbJisYZHoojHqA)

# Protocole de recherche soumis à la commission d’éthique de la recherche clinique de la Faculté de Biologie et Médecine de Lausanne

**1. Titre de l’étude**

Etude randomisée comparant 2 stratégies de dépistage VIH aux urgences par tablette numérique; l’étude *PETTSEQ* (PErformance of Two Testing Strategies by Electronic Questionnaire)

**2. Dates**

Date d’envoi du protocole: 25 mai 2015

Durée de l’étude: août 2015 – décembre 2015

**3. Nom et signature des investigateurs et de leurs collaborateurs:**

Cléo Gillet, étudiante en 1ère année de Master en médecine

Investigateur principal (co-premier auteur)

Dr O. Hugli, PD-MER, MPH, Service des Urgences, CHUV

Tuteur

Dr K.E.A. Darling, MD-PhD, Service des Maladies Infectieuses, CHUV

Cotuteur

Dr M. Cavassini, PD-MER, Service des Maladies infectieuses, CHUV

Cotuteur

**4. Mise en perspective de l’étude**

**4.1 Etat des connaissances actuelles**

Selon UNAIDS, en 2013, environ 20’000 personnes vivaient avec le VIH en Suisse, ce qui correspond à un taux de prévalence de 0,4% chez les adultes de 15 à 49 ans [1]. Les traitements antiviraux combinés actuels permettent de diminuer la virémie à un niveau indétectable chez les patients traités, ce qui a permis de diminuer la mortalité liée au SIDA, ainsi que la morbidité et la dissémination du virus [2]. Néanmoins, malgré les traitements existants et les campagnes préventives, le VIH-SIDA reste une pandémie et la Suisse a recensé 519 nouvelles déclarations d’infection en 2014 [3]. De ces nouveaux cas, on estime que 30% sont diagnostiqués à un stade avancé de l’infection [4]. Un diagnostique tardif implique non seulement une morbidité et une mortalité plus élevée mais aussi un risque de transmission plus important, le patient n’étant pas conscient de sa condition pendant le délai entre la contamination par le virus et le diagnostic [4].

Concernant le dépistage du VIH, plusieurs approches existent:

- L’approche diagnostique qui concerne les personnes présentant un tableau clinique évocateur;
- L’approche ciblée (*targeted*) qui consiste à concentrer une intervention sur les groupes dits *à risques*, notamment les hommes ayant des relations sexuelles avec des hommes (HSH), les utilisateurs de drogues intraveineuses, les personnes ayant des relations sexuelles non-protégées avec partenaires à risque d’être infectés par le VIH, ou les personnes issues de pays à forte prévalence. Ceci implique que la majorité des personnes touchées par l’épidémie ont des caractéristiques communes;
- L’approche non-ciblée (*non-targeted*) vise à dépister l’entier de la population.

Il existe, pour chacune de ces trois approches, 1) un système *opt-in*, processus actif dans lequel le patient accepte de suivre la recommandation de dépistage du médecin, et 2) un système *opt-out*, dans lequel le patient est informé qu’un dépistage est fait systématiquement, sauf s’il demande explicitement de ne pas être dépisté pour le VIH.

Afin d’améliorer les performances de dépistage du VIH aux USA, les Centres pour le Contrôle et la Prévention des Maladies (CDC) préconise depuis 2006 une approche de dépistage systématique non-ciblé de type opt-out dans toutes les zones où l’infection a une prévalence supérieur à 0,1% [5]. Cette approche cible les sujets de 13 à 64 ans et a pour but de dépister un maximum de personnes, en particulier ceux qui ignorent ou minimisent leur prise de risque de contamination par le HIV.

En Suisse, les recommandations de l’Office Fédéral de la Santé Publique (OFSP) divergent vers une forme moins systématique de dépistage. En effet, en 2007 et 2010, une approche dite Provider-Initiated Counselling and Testing (PICT) a été proposée, où le dépistage ciblé du VIH est effectué suite à un entretien initié par le médecin, et vient s’ajouter au Voluntary Counselling and Testing (VCT), où le test est fait à la demande du patient [6]. En 2013, des précisions concernant l’approche PICT ont été publiées dans le bulletin de l’OFSP. Désormais, trois objectifs doivent être atteints: reconnaître une primo-infection, évoquer un diagnostique de VIH à un stade avancé (SIDA) face à certains symptômes évocateurs, et mener un entretien préventif ainsi qu’un dépistage en cas de suspicion de comportement à risque [7]. Suivant la situation, le test sera soit expressément recommandé, recommandé ou proposé et *devra se faire avec consentement du patient (*système *opt-in)*. Il est également notable que cette publication mentionne pour la 1^ère^ fois les centres d’urgences comme le lieu d’un tel dépistage.

Malgré la publication des recommandations PICT en 2010, le nombre de dépistage fait au CHUV n’a pas augmenté entre 2008 et 2012, en particulier aux urgences [8]. Ceci est lié en partie à une méconnaissance des directives par la majorité des soignants. En effet, une étude menée dans cinq services d’urgences romands a montré que seuls 18% des médecins connaissaient les nouvelles recommandations. Parmi ces derniers, le taux de test VIH effectué n’était d’ailleurs pas plus important. Ceci montre donc qu’un problème d’adhésion aux recommandations s’ajoute à la méconnaissance, deux obstacles majeurs au diagnostic des infections VIH aux urgences [9].

En outre, il s’avère que la population en Suisse romande ne connaît pas la modalité opt-in propre au test VIH. Selon une étude au CHUV, 38% des patients ayant été hospitalisés dans le service d’Orthopédie en 2007 pensaient à tort qu’un test VIH était fait d’office lors des prises de sang pré-opératoires [10]. Parmi ces patients, 96% pensaient qu’une absence d’information sur les résultats du test VIH signifiait que ce dernier était négatif. Finalement, plus de 80% des patients interrogés étaient favorables à un dépistage pré-opératoire systématique (non-ciblé) [10].

A l’étranger, plusieurs études se sont intéressées aux services d’urgences pour y mettre en œuvre un dépistage non-ciblé du VIH. Le choix des urgences est pertinent par plusieurs aspects. Tout d’abord, selon la *Society of Academic Emergency Medicine*, les services d’urgences doivent inclure des services médicaux *préventifs, éducatifs ainsi que curatifs* [11, 12]. Les questionnaires de dépistage aux urgences sont non seulement possibles mais également bien reçus par les patients qui n’estiment pas que cela interfère avec la continuité de leurs soins [13]. Les services d’urgences sont un lieu de choix pour le dépistage pour deux raisons additionnelles. La première est le nombre élevé de patients qui y consultent : 37’934 personnes au CHUV en 2014 (données départementales) et 1,62 millions en Suisse en 2011 [14]. La deuxième raison est la patientèle parfois spécifique des urgences. Il a été démontré que les usagers les plus fréquents des urgences représentent une population vulnérable et à risque d’infection VIH [15]. Une étude rétrospective menée dans les services d’urgences suisses montre que 60% des usagers fréquents ont des problèmes de santé mentale ou des problèmes d’addiction et qu’ils cumulent en moyenne plus de 3 facteurs de risque de vulnérabilité [16]. En Suisse, les travailleuses du sexe sont aussi des usagères fréquentes des urgences, ne connaissant pas toujours d’autres portes d’accès aux soins; elles reconnaissent avoir un faible taux de dépistage VIH malgré un comportement sexuel à risque [17]. Il en va de même des populations migrantes issues de pays à forte endémie, moins bien intégrées dans le système de santé suisse.

Malgré les avantages des interventions aux urgences, il semble que le modèle non-ciblé et opt-out proposé par les CDC entraîne un certain taux de refus, souvent lié au fait que les patients, ne se considérant pas à risque, et dès lors ne comprennent pas l’intervention proposée. Ceci est d’autant plus inquiétant que les patients refusant le test présentent un risque trois fois supérieur d’être des porteurs non diagnostiqués du VIH [18]. Une étude menée cette fois auprès des clients des travailleuses du sexe à Lausanne a montré que jusqu’à 46% d’entre eux n’avaient jamais été dépistés pour le VIH [19]. Parmi les clients qui ne voulaient pas se faire dépister, jusqu’à 31% ont donné comme justification l’absence de risque, malgré le fait que jusqu’à 22% avaient eu plus que 5 partenaires sexuelles pendant les 2 ans précédents et que jusqu’à 32% avaient eu des rapports vaginaux et anaux non protégés [19]. Cette étude suggère que des questions sur les types de risque pris amèneraient un taux de dépistage plus élevé qu’une simple invitation au dépistage chez certaines personnes potentiellement à risque d’infection VIH.

En effet, une approche plus interactive informant le patient sur les risques d’infection au VIH permet une meilleure auto-évaluation des comportements à risque et pourrait améliorer le taux de dépistage VIH [20]. Pour proposer une telle approche, un dépistage aux urgences par un moyen électronique s’avère particulièrement bien accepté par les patients [13]. Les avantages sont en effet nombreux: sentiment d’anonymat pour le patient face à une tablette numérique, adaptation possible à sa langue et peu d’investissement nécessaire de la part du personnel soignant [13]. Les nouvelles technologies permettent aussi de gagner du temps et de faciliter une approche individuelle du patient, en permettant de proposer des questionnaires interactifs qui intègrent de manière dynamique les caractéristiques du patient et ses réponses pour proposer des questions plus pertinentes[21]. Cette approche parvient alors à augmenter l’attention du patient plus efficacement que par des méthodes standardisées non interactives [21]. De plus, le comportement à risque des patients ayant reçu de telles interventions peut être réduit à long terme [22], les personnes se rappelant mieux d’avoir reçu des conseils pour améliorer leur santé [23].

Dans le cadre spécifique du VIH, les comportements à risque sont plus fréquemment rapportés lorsque les patients sont face à une plateforme électronique que face à un soignant [24]. Cela s’explique en partie par le fait que les groupes les plus à risque pour une infection au VIH sont très stigmatisés et sont donc plus à l’aise de rapporter d’éventuelles prises de risques sur une tablette par définition neutre [25].

Une étude menée aux Etats Unis, en utilisant des interfaces électroniques aux urgences pour évaluer les facteurs de risque des patients, montre que 71% des patients préfèrent se soumettre à un questionnaire électronique contre 18% à des questions d’un un soignant. Cette étude a aussi montré que l’interface électronique permettait d’identifier un pourcentage plus élevé de patients candidats au dépistage VIH, mais que le pourcentage de ceux qui acceptait de faire le test recommandé électroniquement était plus faible que lorsque cette offre était faite par un soignant. Le bilan net était donc une parité entre les deux approches [26]. Le dépistage du VIH par un soutien électronique aux urgences est donc possible, bien accepté par les patients et permet de dépister plus de personnes ayant un comportement à risque. Ces interventions permettent aussi d’assurer une prise en charge rapide des patients nouvellement diagnostiqués, tout en permettant de faire passer un message de prévention aux personnes se soumettant au dépistage [27].

Jusqu’à présent, il n’y a pas de donnée en Suisse sur la faisabilité de l’utilisation des moyens électroniques pour optimiser le dépistage VIH aux urgences. Un outil électronique serait facile à utiliser par des patients attendant des soins et serait également un moyen de contourner des obstacles au dépistage VIH décrits dans la littérature tel que l’absence fréquente de confidentialité lors d’interview par du personnel médical aux urgences, et surtout le manque de temps et d’envie des soignants pour aborder ce sujet sensible [28]. Il n’y a en plus pas de donnée actuellement en Suisse concernant la méthode de dépistage la plus efficace – ciblée versus non-ciblée – spécifiquement auprès des patients qui se présentent aux urgences.

**4.2 Étude Proposée**

La question posée est de savoir si le taux de dépistage au moyen d’une tablette électronique est meilleure avec un questionnaire électronique auto-administré ciblant les facteurs de risque VIH du patient qu’avec une proposition de dépistage systématique (non-ciblé) sans questionnaire préalable.

Si l’approche suisse, *i.e.* une approche diagnostique ciblée et opt-in, semble insuffisante, les études testant les recommandations des CDC aux urgences, c’est-à-dire un dépistage non-ciblé en opt-out ont montré un plus haut taux de refus que par une approche ciblée. Cependant, ce taux de refus plus élevé est compensé par le plus grand nombre de personnes à qui le dépistage est proposé, et avec au plus de nouveaux diagnostics d’infection VIH par rapport à une approche ciblée [29].

Nous proposons donc de comparer le taux de dépistage par test VIH rapide en mode opt-in obtenu par une approche non-ciblée à celui obtenu par un questionnaire ciblant les facteurs de risque du patient, tous deux auto-administrés via une tablette électronique. Ce questionnaire (voir 7.2.2) concernera les possibles comportements à risque du patient selon les recommandations de l’OFSP et orientera le patient selon ses réponses vers un test VIH ou non. Nous appliquons donc par cette étude le troisième point des recommandations de l’OFSP *évaluation des comportements à risque*. Ceci a une importance non seulement clinique mais également médico-légale puisqu’un avis de droit a conclu à la responsabilité médico-légale du médecin en cas de non respect de ces recommandations [7].

Aux urgences du CHUV dans une étude effectuée en 2013, 28% de 411 patients ont accepté d’être dépistés de manière systématique (non-ciblé) (Favre-Bulle *et al*., manuscrit soumis). A l’inverse, dans une étude menée aux Etats-Unis au moyen d’un questionnaire auto-administré par tablette électronique ciblant les facteurs de risque VIH du patient, une telle intervention a amené une prise de conscience de comportements à risque chez 11% des patients [30]. Sur la base de ces deux études menées dans des contextes et avec des moyens différents, l’approche systématique semble donc supérieure. Notre étude permettra de vérifier si, en Suisse, l’approche systématique est également supérieur à une approche ciblée quand toutes les deux sont réalisées au moyen d’une tablette numérique et adoptent une approche opt-in.

**4.3 Buts de l’étude**

Hypothèses :

- Le taux de réalisation du dépistage VIH par une approche systématique (*i.e.* non-ciblée et opt-in) sera supérieur à une approche ciblée et opt-in basée sur les facteurs de risques définis par l’OFSP.
- Chez les patients refusant un dépistage systématique, une augmentation du taux de réalisation du dépistage VIH sera observée après une auto-évaluation des facteurs de risques par tablette électronique.
- Les 2 approches amèneront une augmentation des tests VIH effectués aux urgences du CHUV par rapport à 2008-2012 [8].
- Dans la population participant à l’étude, l’usage autonome de la tablette électronique permettra un auto-administration complète du questionnaire pour plus de 80% des patients.

**4.4 Objectifs et justification**

*Objectifs*

- Objectif I^aire^ : comparer le taux de dépistage par test rapide VIH chez les personnes ayant préalablement reçu un questionnaire d’évaluation de comportement à risque, par rapport à un groupe témoin ayant reçu la proposition d’un dépistage VIH systématique (Figure 1).
- Objectifs II^aires^ :
  - évaluer le taux de dépistage VIH.
  - évaluer la facilité d’utilisation de la tablette électronique dans le cadre des urgences.
  - comparer les caractéristiques démographiques des patients acceptant ou non le test VIH.

*Justifications*

- Au vue des opportunités manquées aux urgences tant par les médecins n’appliquant pas les directives de l’OFSP que par les patients croyant à un dépistage systématique lors de prise de sang, le dépistage du VIH est insatisfaisant. Cette problématique n’est pas anodine puisqu’un pourcentage non négligeable de la population, en particulier la population jeune et migrante n’est pas suivie par un médecin de premier recours. Le passage aux urgences représente donc la seule opportunité de dépistage, en particulier à un stade précoce de l’infection [31]. Il est donc nécessaire d’instaurer et pérenniser un système permettant de toucher un maximum de patients et de diagnostiquer une infection VIH au stade le plus précoce possible. A l’époque des thérapies anti-virales hautement efficaces, le diagnostic précoce des personnes infectées par le VIH permet non seulement de réduire leur mortalité, morbidité et le risque de transmission du virus, mais réduit les coûts du traitement.

De manière plus générale, si l’utilisation de tablette s’avérait facile pour les patients aux urgences, il serait alors possible d’étendre leur application à d’autres interventions de dépistage ou de prévention, ajoutant un bénéfice à la consultation qui irait au-delà du simple motif de recours initial.

**5. Plan général de l’étude**

Etude randomisée en simple aveugle (investigatrice ignorante du bras de l’étude auquel le patient est randomisé), se faisant par l’intermédiaire d’une tablette électronique. Elle se déroulera dans le service des urgences du CHUV entre août et décembre 2015 et est inscrite dans le cadre du travail de maîtrise universitaire en médecine.

**6. Sélection des sujets**

Nous cherchons à inclure 160 patients des urgences du CHUV dans cette étude.

Critères d’inclusion :

- Patient ≥ 18 ans et ≤ 75 ans
- Patient admis durant les 12 dernières heures

Critères d’exclusion (Annexe 1):

- Patient instable cliniquement (passage en salle de réanimation ou jugé comme tel par le médecin en charge du cas)
- Patients transférés d’un autre hôpital
- Patient incapable de discernement ou avec qui il est impossible de communiquer  (à cause de, par exemple, troubles cognitifs [démence], retard mental, éthylisation aiguë au moment du passage de l’investigatrice, intoxication aux opiacés ou autres substances psycho-actives, psychose aiguë, ou patient malentendant ou non francophone)
  - - Patient(e) connu(e) comme VIH+
    - Patient ayant déjà eu une proposition de dépistage VIH aux urgences par le médecin en charge du patient avant le passage de l’investigatrice
    - Patient incarcéré

**7. Déroulement de l'étude et investigations prévues**

**7.1 Lieu et durée de l’étude**

L’étude se déroulera au service des Urgences du CHUV entre août et décembre 2015. La récolte des données se fera tous les jours de la semaine entre 8h et 20h, afin de s’assurer qu’un médecin référent soit toujours présent pour prendre en charge un patient de l’étude au cas où le test rapide de dépistage VIH s’avérait positif (voir 7.2.2., ci-dessous). Le délai de 12h entre admission et passage de l’investigatrice permettra d’inclure également les patients admis sur la nuit.

Les patients seront approchés par l’investigatrice dans leur *box de consultation*, où ils attendent généralement entre 10 et 40 minutes avant le 1^er^ contact avec le médecin, et plusieurs heures avant la fin de la consultation. La confidentialité de l’entretien entre l’investigatrice et le patient sera ainsi garantie.

*Parcours d’un patient consultant aux urgences du CHUV*


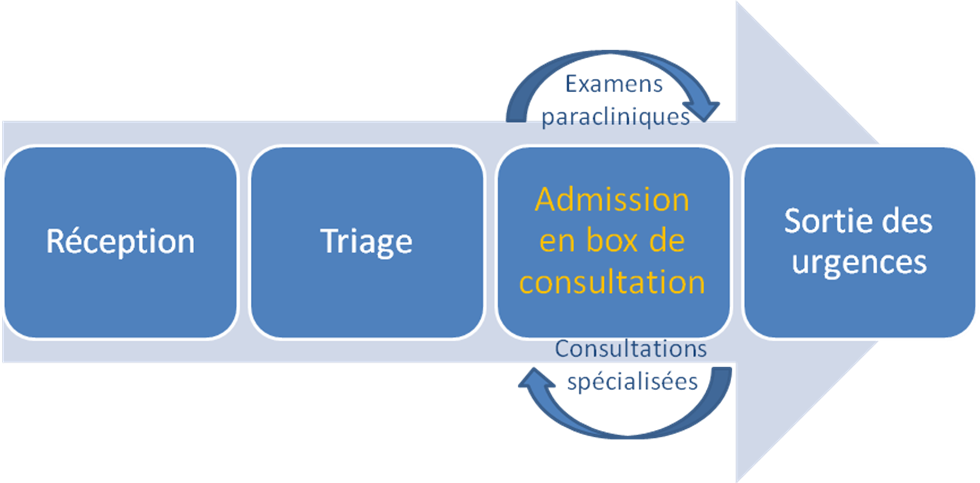


**7.2 Déroulement de l’étude**

**7.2.1. Formation de l’investigatrice et information au personnel soignant**

L’investigatrice sera formée à effectuer le test rapide VIH (anticorps anti-VIH), utilisant le test INSTI™ qui requiert une prise de sang capillaire au bout du doigt du patient. Le personnel soignant des urgences sera informé du déroulement de l’étude lors de l’un de leurs colloques.

**7.2.2. Procédure** (Figure 1)

L’investigatrice sélectionnera les patients enregistrés aux urgences éligibles pour l’étude sur la base des informations du dossier médical. Après avoir vérifié qu’aucun critère d’exclusion n’est applicable (Annexe 1), les patients seront randomisés en proportion 1:1 entre les 2 bras de l’étude. La randomisation se fera par bloc de permutation de taille de 4 ou 6 répartis aléatoirement. La liste d’attribution aux groupes sera générée à l’aide du site [www.randomization.com](http://www.randomization.com/). L’appartenance au groupe sera inscrite sur un billet dans une enveloppe opaque. Les enveloppes seront numérotées consécutivement et ouvertes par l’investigatrice dans l’ordre de numérotation au moment de la randomisation. Afin d’éviter un biais induit par la présentation détaillée des deux stratégies de dépistage avant remplissage du questionnaire par le patient, les étapes d’’information détaillée sur l’étude et obtention de l’accord signé seront effectuées une fois le questionnaire rempli ou l’information présentée sur tablette numérique et la proposition de test faite (Annexe 2 et Figure 1). L’étudiante se présentera au patient et lui proposera de participer à une étude sur le dépistage du VIH aux urgences. Les termes de ce message oral sont écrits dans l’Annexe 8. En cas de refus de participer à l’étude, seules les données démographiques de base seront enregistrées sans aucun identifiant (âge, sexe), ceci afin de permettre de comparer les participants aux non participants et donc exclure un biais de sélection. En cas de refus de participer après avoir répondu au questionnaire, les réponses et la décision regardant le test de dépistage ne seront pas inclus dans l’analyse finale de l’étude. Seules les données démographiques de base sans identifiant seront enregistrées (âge, sexe).

Deux groupes  de patients inclus seront ainsi formés:

1. Ceux recevant un questionnaire sur tablette électronique, basé sur les critères du dépistage VIH de l’OFSP. Après remplissage du questionnaire, un test rapide sera offert si le résultat du questionnaire le recommande. Il est nécessaire de souligner que l’investigatrice aura uniquement accès à la recommandation finale du questionnaire et ne verra donc pas les réponses du patient.
2. Ceux recevant la proposition sur tablette électronique d’un dépistage VIH systématique après une brève information sur la pertinence d’une telle stratégie (Annexe 3).

Si le test VIH est accepté, et un consentement additionnel signé (Annexe 4), il sera effectué par l’investigatrice durant sa consultation aux urgences. Les sujets du groupe 1 ci-dessus, qui n’ont aucun facteur de risque de VIH selon le questionnaire (Annexe 5), recevront une proposition sur tablette de faire un test VIH malgré tout, leur permettant ainsi de profiter de leur consultation pour bénéficier d’un dépistage ; les individus du groupe 2 qui refusent un dépistage VIH non-ciblé seront invités à compléter le questionnaire ciblé reçu par le groupe 1. Si le questionnaire amène à une recommandation de dépistage en raison de leur profil de risque, ils pourront alors effectuer le test VIH.

Tous les patients inclus recevront, après le questionnaire par tablette, un questionnaire concernant leur niveau socio-économique, leur orientation sexuelle et s’ils ont un médecin traitant (Annexe 6). Ces données, ainsi que leurs données démographiques obtenues d’Axya permettront l’analyse des caractéristiques des patients ayant accepté le dépistage et de ceux l’ayant refusé. Les patients seront invités également à donner leur avis sur la facilité de l’utilisation de la tablette et sur l’opportunité d’offrir un dépistage d’urgences (Annexe 6). Par ailleurs, l’investigatrice restera à proximité immédiate du box pour répondre à toute demande d’assistance du patient, que ce soit pour utiliser la tablette que pour répondre à des questions concernant les items du questionnaire.

L’investigatrice utilisera un dernier questionnaire sur la tablette permettant d’entrer premièrement si le test a été accepté ou refusé, et deuxièmement le résultat du test s’il a été fait. Il sera aussi possible d’entrer les raisons d’un potentiel refus de dépistage (Annexe 7).

Si le test rapide s’avère *réactif* (positif ou douteux), l’investigatrice en informera le médecin en charge du patient. Ce dernier informera le patient du résultat et lui expliquera qu’une confirmation par prise de sang est nécessaire. Le médecin en charge du patient ordonnera le test de confirmation et avertira immédiatement le médecin de la consultation VIH, ou le médecin de garde en infectiologie pour une consultation en urgence et organisation du suivi en consultation ambulatoire d’infectiologie au cas où le test de confirmation s’avérait positif.

Pour les patients refusant le test rapide, l’intervention sera complétée par des prospectus sur la prévention des comportements à risques, ainsi que les lieux de dépistages existant dans la région.

**Figure 1.** Schéma du protocole de l’étude

**
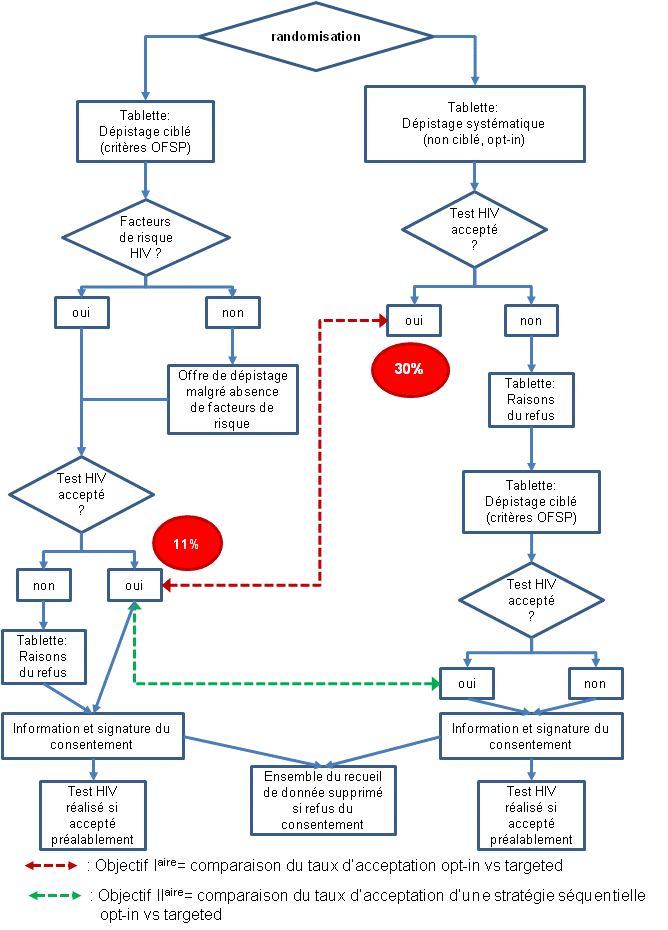
**

**7.2.3. Questionnaire Informatique**

Le questionnaire sera généré grâce à l’outil mis à disposition par le site SurveyMonkey, site régulièrement utilisé au CHUV pour des sondages. Pour garantir l’anonymat des données potentiellement sensibles hébergées sur un site extramuros, aucun identifiant ne sera recueilli sur ce site. Le questionnaire online sera porteur d’un identifiant unique pour chaque patient et propre à l’étude. L’investigatrice notera cet identifiant sur le questionnaire papier. Pour garantir le respect de l’anonymat des sujets lors de l’analyse, l’investigatrice analysera les résultats à partir d’une base de données qui concaténera les résultats issus du site de SurveyMonkey, d’Axya et des documents papiers et dont tous les identifiants (IPP, N° de séjour, nom du patient) auront été supprimés par les responsables de l’étude.

- 1. **Paramètres enregistrés**

1. Caractéristiques démographiques du patient (sur la base des données hospitalières et sur la base d’un questionnaire papier, Annexes 1 et 5):
   - Âge, sexe, origine, état civil, niveau d’éducation
2. Caractéristiques médicales du patient (sur la base des données hospitalières et sur la base d’un questionnaire papier):

- Motif de présentation
- Diagnostic à la fin de la consultation aux urgences
- Type de traitement proposé
- Médecin traitant (oui/non)

1. Dépistage VIH (sur tablette informatique)

- Proposé
- Accepté
- Effectué
- Résultat (si effectué)

1. Facteurs de risque VIH non détaillés dans les recommandations de l’OFSP :
   - Leur orientation sexuelle (sur la base d’un questionnaire papier, Annexe 5)
2. Raison(s) du refus si test non effectué (sur la base d’un questionnaire en annexe inséré dans le questionnaire informatique)

- Impression de ne pas être à risque pour le VIH
- test VIH récemment effectué et négatif et sans facteur de risque depuis
- Pas de temps d’effectuer un test aux urgences
- Préférence d’effectuer le test chez le médecin traitant
- Préférence d’effectuer le test dans un centre de dépistage anonyme
- Peur du résultat
- Peur que la famille apprenne le résultat
- Peur que l’employeur apprenne le résultat
- Autre : (texte libre)

**7.4 Analyse statistique**

Sur l’hypothèse que la proposition d’un dépistage VIH non-ciblé opt-in va augmenter le taux de dépistage de ~19% par rapport à un questionnaire sur les facteurs de risque du patient, 80 participants par groupe (dépistage non-ciblé opt-in et dépistage ciblé selon les critères de l’OFSP) sont nécessaires pour avoir une puissance de 80% de démontrer cette différence, avec un risque d’erreur de type I fixé à 5%.

**8. Surveillance médicale**

Non applicable

**9. Rôle du personnel infirmier**

Non applicable

**10. Médicaments**

Non applicable

**11. Évaluation des risques**

Cette étude propose un test rapide (INSTI™) ne suffisant pas à poser le diagnostique d’infection VIH. Il doit être couplé à un test VIH standard en laboratoire. La probabilité de faux positif avec le test rapide utilisé dans cette étude est extrêmement faible: 1/9072 selon une étude française multicentrique [32]. Les participants seront informés de ce risque du test grâce à un formulaire d’information qui fait partie du consentement pré-test rapide pour VIH.

Dans le cas d’une exposition remontant à moins de 12 semaines, l’investigatrice précisera qu’un test négatif n’exclut pas une primo-infection, et qu’un test rapide doit être répété 12 semaines après la dernière exposition.

Dans le cas d’un résultat *réactif* (positif ou douteux), l’investigatrice informera le médecin en charge. Ce dernier en informera un/une des chef(fe)s de clinique de médecine 2, puis effectuera un test au laboratoire d’immunologie du CHUV en urgence. Un résultat définitif sera obtenu dans les 2 heures. Il est à relever toutefois que la probabilité d’un test réactif dans le cadre de ce travail est très faible : sur la base de la prévalence de séropositivité en Suisse de 0.4% et en postulant qu’un tiers des patients ignorent leur séropositivité, cette probabilité est d’environ 0.2% pour notre collectif de 160 cas.

Si le test de confirmation est négatif, le médecin en charge fera directement l’annonce au patient. Si le test de confirmation est positif, l’annonce se fera par le médecin en charge qui sera accompagné par un/une des chef(fe)s de clinique de médecine 2.

Le déroulement de l’étude entre 8H et 20H assure la présence d’un spécialiste VIH afin d’assurer une prise en charge optimale du patient, si son test s’avère être positif.

À partir du diagnostique confirmé, un traitement pourra être initié dès que possible, permettant ainsi d’améliorer son pronostic et de minimiser les risques de transmission à ses partenaires sexuels.

Il est à noter qu’une étude est en cours en ce moment au CHUV : ***Motivation et acceptation du test VIH aux urgences: enquête auprès des médecins et des patients***, menée par deux étudiants en deuxième année de Master. Elle implique aussi l’utilisation de tests rapides administrés par les étudiants investigateurs (Protocole N°95/14). La phase de collecte de donnée s’est déroulée sans complications après inclusion de 100 patients aux urgences du CHUV.

**12. Couverture d'assurance**

En cas de dommages éventuels causés aux participants, le CHUV répondra de ces derniers en sa qualité de promoteur conformément aux dispositions légales applicables. .

**13. Formulaires d'information et de consentement**

Attachés en pièce ci-jointe

**14. Traitement de données personnelles et d'échantillons biologiques**

Les données personnelles des patients seront traitées par l’investigatrice (Cléo Gillet) de façon confidentielle et anonymisée. Elles seront introduites de manière anonyme dans une base de données Excel. Celle-ci ne contiendra aucun identifiant ou information permettant d’identifier la personne source. Le questionnaire électronique utilisera le site *SurveyMonkey* et ne contiendra aucune question relative à l’identité du patient.

**15. Plan de financement et rétribution**

Les frais engendrés par cette étude seront couverts par la faculté de biologie et médecine selon les défraiements alloués pour les travaux de Master ainsi que le fond de recherche du Dr. Cavassini ou sur le fond de recherche des urgences. Des frais engendrés en cas d’un test rapide VIH *réactif* (positif ou douteux) seront pris en charge par la caisse maladie du patient.

**16. Étude impliquant la participation de praticiens installés**

Non applicable

**17. Information au personnel soignant médical et paramédical**

L’information sur le déroulement de l’étude se fera auprès des infirmiers et des aides-infirmiers lors de leur colloque d’équipe mensuel, selon ce qui se pratique dans le service des urgences.

**18. Coûts**

Les dépistages VIH effectués sur l’initiative du médecin selon le PICT sont facturés à l’assurance maladie. Les tests à lecture rapide effectués par l’investigatrice (étudiante master) ne seront pas facturés au patient. Les tests de confirmation seront toujours facturés à l’assurance maladie du patient.

CHF 9,50 par test à lecture rapide: CHF 1,520- (maximum, avec un taux de dépistage à 100% des participants)

Salaire de KD à 10% durant 8 mois:  CHF 8000-

Tablette numérique: CHF 300

Site *SurveyMonkey*: CHF 450 CHF

**Références**
