# Supplementary material for: Targeted versus non-targeted HIV testing offered via electronic questionnaire in a Swiss emergency department: A randomized controlled study
Source: PLoS One. 2018 Mar 7;13(3):e0190767. doi: 10.1371/journal.pone.0190767 (PMC5841645; doi:10.1371/journal.pone.0190767)
Supplement: S2 Text — (DOC) [file pone.0190767.s003.doc]

# [
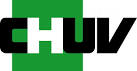
](http://www.google.ch/url?q=http://www.cliniquedudos.ch/fr/cv.html&sa=U&ei=BmAwU_z2Duiy7Ab2q4CoBg&ved=0CC8Q9QEwAQ&usg=AFQjCNHJiHnIRTgaIj_KqBXVvvkc3FrczA) [
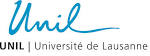
](http://www.google.ch/url?q=http://ceat.epfl.ch/&sa=U&ei=XWAwU-H8GY6rhAforoHQCA&ved=0CC8Q9QEwAQ&usg=AFQjCNE7XSjB3ccF0LlRcbJisYZHoojHqA)

# Research proposal for submission to the Clinical Research Ethics Committee of the Faculty of Biology and Medicine, University of Lausanne

**1. Study title**

Randomised study comparing two HIV screening strategies in the Emergency Department using an electronic tablet questionnaire: The *PETTSEQ* study (PErformance of Two Testing Strategies by Electronic Questionnaire)

**2. Dates**

Date of proposal submission: 25 May 2015

Study duration: August 2015 – December 2015

**3. Name and signature of the investigators and collaborators:**

Cléo Gillet, medical student, first year of Masters

Principal investigator (joint first author)

Dr O. Hugli, PD-MER, MPH, Consultant, Emergency Department, Lausanne University Hospital (LUH)

Tutor

Dr K.E.A. Darling, MD-PhD, Consultant, Infectious Diseases Service, LUH

Co-tutor

Dr M. Cavassini, PD-MER, Consultant, Infectious Diseases Service, LUH

Co-tutor

**4. Background**

**4.1 State of current knowledge**

According to UNAIDS, in 2013, around 20,000 people were living with HIV in Switzerland, equivalent to a seroprevalence rate of 0.4% among adults aged between 15 and 49 years old [1]. In treated patients, current combined antiviral regimens enable reduction of the viral load to undetectable levels, with associated reduction in AIDS-related morbidity, mortality and onward transmission of the virus [2]. Despite existing treatments and prevention campaigns, the HIV-AIDS pandemic continues and Switzerland reported 519 new infections in 2014 [3]. Among these new cases, roughly 30% are diagnosed at an advanced stage of infection [4]. Late diagnosis results not only in increased morbidity and mortality but also in increased risk of transmission, as the individual is unaware of being infected throughout the time between becoming HIV positive and being diagnosed [4].

Three broad HIV-screening approaches exist:

- *Diagnostic* testing concerns individuals who present with symptoms or signs suggestive of HIV infection;
- *Targeted* testing focuses on groups at risk, notably men who have sex with men (MSM), injecting drug users, individuals from countries of high HIV seroprevalence, and individuals having unprotected sex with partners from groups at risk of HIV infection. This approach assumes that most people affected by HIV will have common characteristics;
- *Non-targeted* screening offers screening to all individuals within a population, regardless of HIV infection risk factors.

For each of these three approaches, the testing can be 1) *opt-in*, where the patient actively accepts testing when offered, and 2) *opt-out*, where the patient is informed that testing will take place unless he or she explicitly refuses.

To improve HIV testing rates in the US, the Centers for Disease Control and Prevention (CDC) has recommended non-targeted opt-out testing since 2006 in all areas where HIV prevalence is greater than 0.1% [5]. This approach targets individuals aged 13-64 years old and aims to maximize screening, particularly among those unaware that they may be at risk of infection.

In Switzerland, the Federal Office of Public Health (FOPH) recommends diagnostic and targeted testing rather than non-targeted screening. In 2007 and 2010, the FOPH recommendations proposed Provider-Initiated Counselling and Testing (PICT). With PICT, targeted HIV testing is performed upon the initiative of the doctor, as opposed to Voluntary Counselling and Testing (VCT), where testing is performed at the request of the patient [6]. In 2013, updated recommendations expanded the PICT approach, emphasising three objectives: to recognise primary (acute) HIV infection, to recognise symptoms/signs associated with advanced HIV infection (AIDS), and to conduct an interview on HIV risk and prevention and offer screening in the case of risk behaviour [7]. According to the situation, HIV testing should be expressly recommended, recommended or proposed and must be performed with the consent of the patient (*opt-in*). In the 2013 FOPH recommendations, the Emergency Department (ED) is mentioned specifically as a place in which HIV testing should occur.

In spite of the 2010 PICT recommendations, the HIV testing rate at LUH did not increase between 2008 and 2012, particularly in the ED [8]. In the ED, this was partly explained by ED doctors being unaware of the FOPH testing recommendations. Among ED doctors in the EDs of the five main teaching hospitals in French-speaking Switzerland, only 18% were aware of the FOPH recommendations. Further, awareness was not associated with performing more HIV tests. In this way, being unaware of the recommendations and failing to adhere to them are two major obstacles to diagnosing new HIV infections in the ED setting [9].

From the patient side, many patients in French-speaking Switzerland are unaware of the opt-in requirement for HIV testing, whereby patients cannot be tested for HIV without first giving consent. In a study conducted at LUH, 38% of patients admitted for elective orthopaedic surgery in 2007 believed erroneously that they had been tested for HIV as part of their pre-operative work-up [10]. Of patients with this belief, 96% assumed that the lack of information about their HIV test (which they never had) meant that the test was negative. Of all patients questioned, over 80% were in favour in principle of routine (non-targeted) pre-operative HIV screening [10].

Elsewhere, several studies have examined the role of the ED as a site for non-targeted HIV screening, the ED being a potential site for several reasons. First, according to the *Society of Academic Emergency Medicine*, the ED should provide medical services for prevention and education as well as for treatment [11, 12]. Questionnaires in the ED on screening are not only possible to conduct but they are well-received by patients who do not find that they interfere with delivery of health care [13]. The ED is suitable for screening for two further reasons. First, the ED has a high patient turnover : in 2014, 37,934 patients presented to LUH ED (departmental data) and 1.62 million patients presented to the ED in Switzerland overall [14]. Second, ED patients sometimes represent distinct socioeconomic groups. Frequent users of the ED represent a population which is both vulnerable and at risk of HIV infection [15]. A retrospective study conducted in EDs in Switzerland showed that 60% of frequent users had mental health or addiction problems and presented a mean of more than three vulnerability risk factors [16]. In Switzerland, sex workers are also frequent users of the ED as they are unaware of other services at which they can access health care; the HIV testing rate in this group is often low despite high risk sexual behaviour [17]. Migrant populations from countries of high HIV seroprevalence are often less well integrated into the Swiss health care system.

Despite advantages of testing interventions in the ED, it seems that the non-targeted, opt-out model proposed by the CDC is not universally adopted in the US, often linked to the fact that patients do not consider themselves at risk and so do not take up the offer of testing. This is concerning when patients declining testing often present a high risk of having undiagnosed HIV infection [18]. One study conducted among the clients of sex workers in Lausanne reported that 46% had never been tested for HIV [19]. Among clients who declined to be tested, 31% gave the reason of not being at risk, despite the fact that 22% reported having >5 sexual partners during the previous two years and that 32% reported unprotected vaginal and anal sex [19]. This study suggests that, among individuals potentially at risk of HIV, questions focusing on the types of risk taken would increase HIV testing rates over the simple offer of testing.

Indeed, a more interactive approach informing the patient of HIV risk allows a better self-assessment of risk behaviour which would in turn increase HIV testing uptake [20]. To adopt such an approach, offering testing in the ED by way of electronic tablets is well-accepted by patients [13]. There are numerous advantages: the feeling of anonymity for the patient with an electronic tablet; the possibility of offering information / testing in the appropriate language; and minimal time investment required from treating health care personnel [13]. New technologies save time and can individualise each approach to the patient taking part, using interactive questionnaires which integrate patient characteristics and questionnaire responses and then adapt the questionnaire to asking the most relevant questions [21]. This dynamic approach increases patient attention compared to traditional, non-interactive methods [21]. Further, risk behaviour in patients who have taken part in such interventions may reduce over the long-term [22], as these individuals remember the advice they received for improving their health [23].

In the specific context of HIV, risk behaviour is more frequently reported by patients when faced with an electronic device than when faced with a health care provider [24]. This is partly explained by the fact that at-risk groups are highly stigmatized and are more comfortable relaying risk information to an electronic tablet which is passive and neutral [25].

A study conducted among ED patients in the US, using electronic questionnaires to determine patient risk factors, reported that 71% of patients preferred responding to an electronic questionnaire while 18% preferred responding to a health care worker. This study also showed that the electronic questionnaire identified a higher percentage of patients eligible for HIV testing but that the percentage of patients accepting the offer of testing by the electronic tablet was lower than that among patients offered testing by a health care worker. The final result thus showed no difference in testing rates between the two approaches, electronic versus human [26]. HIV screening using electronic tablets is thus feasible, well-accepted by patients and allows testing of patients with risk behaviour. Such interventions also enable rapid engagement in care of newly-diagnosed patients while transmitting a message of prevention to individuals who agree to screening [27].

To date, there are no data in Switzerland on the feasibility of electronic tablets in optimising HIV testing in the ED. An electronic tablet would be easy to use by patients who are waiting to be seen in their ED cubicle and would provide a means of bypassing obstacles to HIV testing which have been described, such as lack of confidentiality, lack of time and lack of motivation among ED doctors to broach this sensitive subject [28]. There are also no data in Switzerland demonstrating the optimal HIV screening method – targeted versus non-targeted – specifically in the ED setting.

**4.2 Proposed study**

The study aims to determine whether the HIV testing rate achieved using electronic tablets is better with a self-administered electronic questionnaire targeting HIV risk factors than with the offer of non-targeted screening without an initial questionnaire.

If the Swiss approach, that is,

If the Swiss HIV testing approach, targeted and opt-in, appears suboptimal, studies examining testing uptake in the ED with the CDC approach, non-targeted and opt-out, describe a higher rate of patient refusal with the CDC approach than with the targeted approach. However, the higher refusal rate is compensated by the higher number of individuals who are offered testing, and by the greater number of new HIV diagnoses made compared to the targeted approach [29].

We therefore aim to compare the testing rate using rapid HIV testing according to two approaches, each using an electronic tablet: non-targeted opt-in screening compared to testing following a questionnaire identifying patient risk factors.

The questionnaire (see 7.2.2) covers potential risk behaviours of the patient, according to the FOPH HIV testing recommendations, and directs the patient towards HIV testing or not, depending on his/her responses. This study therefore applies the third objective of the FOPH recommendations, to assess risk behavior. This is important not only clinically but also medico-legally as it is now stipulated in the FOPH testing recommendations that non-adherence to these recommendations constitutes a penal offense [7].

In a study conducted in the LUH ED in 2013, 28% of 411 patients agreed to be tested after the offer of non-targeted testing (Favre-Bulle *et al*). Conversely, in a US study using a self-administered questionnaire targeting patient HIV risk factors, such an intervention demonstrated risk behavior in 11% of patients [30]. On the basis of these two studies conducted in different situations and using different methods, the non-targeted approach seems to be superior. Our study will enable us to determine if, in Switzerland, the non-targeted approach is as effective as the targeted approach when both are offered using an electronic tablet and opt-in testing.

**4.3 Study aims**

Hypotheses:

- The HIV testing rate resulting from non-targeted opt-in screening will be higher than that resulting from targeted testing based on risk factors as defined by the FOPH.
- Among patients declining non-targeted screening, the HIV testing rate will increase following self-assessment of risk factors using an electronic tablet.
- The two approaches (non-targeted and targeted) will increase the HIV testing rate compared to that observed at LUH during the period 2008-2012 [8].
- Among patients participating in the study, independent use of the electronic tablet will enable self-administration of the questionnaire for >80% of patients.

**4.4 Objectives and justification**

*Objectives*

- Primary Objective: to compare testing rates, using rapid HIV testing, between patients initially receiving a questionnaire assessing HIV risk factors (targeted testing) and those receiving an offer of non-targeted HIV screening directly without prior assessment (Figure 1).
- Secondary Objectives:
  - To determine HIV screening rates;
  - To determine ease of use of electronic tablets among ED patients;
  - To compare the demographic characteristics of patients accepting or declining HIV testing.

*Justification*

Missed opportunities for HIV testing occur in the ED, through doctors being unaware of or not adhering to the FOPH HIV testing recommendations and through patients believing erroneously that they are routinely tested for HIV when they have a blood test, making HIV screening rates unsatisfactory. This is a problem when a non-negligible proportion of patients presenting to the ED, notably young patients and migrants, are not under a general practitioner. For these patients, the ED visit may represent the only opportunity for HIV screening, in particular during early stages of infection when the individual may be asymptomatic [31]. It is therefore necessary to introduce and maintain an HIV screening approach which reaches a maximum number of patients and which diagnosis HIV at the earliest possible stage. With highly-active anti-retroviral therapy, early diagnosis of HIV reduces not only morbidity, mortality and risk of onward transmission, but also the cost of treatment.

More generally, if the use of electronic tablets is feasible and acceptable to ED patients, it would be possible to extend their use to other screening or prevention interventions, providing a benefit to the ED visit in addition to the treatment received for the reason for presenting.

**5. Study plan**

A randomised, single-blind study (the study investigator will not know which arm each patient is assigned to) conducted using an electronic tablet. The study will take place in the LUH ED between August and December 2015 in the context of the Masters project of a medical student studying at the Faculty of Medicine at Lausanne University.

**6. Selection of participants**

We aim to include 160 patients from LUH ED.

Inclusion criteria:

- Patients ≥ 18 and ≤ 75 years old
- Patients admitted to an ED examination cubicle within the preceding 12 hours

Exclusion criteria (Annex 1):

- Patients clinically unstable (admitted to resus or considered unstable by the treating ED doctor)
- Patients transferred from another hospital
- Patients unable to provide informed consent  (for example, through cognitive impairment, acute alcohol intoxication or intoxication by opiates or other psycho-active substances, acute psychosis, being hard of hearing or not French-speaking and with no interpreter)
  - - Patients of known HIV+ status
    - Patients who have already been offered HIV screening during their ED visit by the treating ED doctor prior to enrolment in the study
    - Prisoners

**7. Study protocol and planned investigations**

**7.1 Study site and duration**

The study will take place in the ED of LUH between August and December 2015. Data will be recorded every day between 08:00H and 20:00H to ensure the presence of an HIV specialist for referral in the event of a reactive rapid HIV test (see 7.2.2., below). The limit of 12 hours prior to study inclusion enables patients admitted during the night to be included in the study.

The patients will be approached by the study investigator in the consultation cubicle, where they generally wait for between 10-40 minutes before their first contact with the ED doctor and several hours before the end of the consultation. Confidentiality during the patient-investigator interview will be guaranteed as all questionnaire questions are read by the patient from the electronic tablet rather than spoken out loud.

*Patient pathway during a visit to LUH ED*


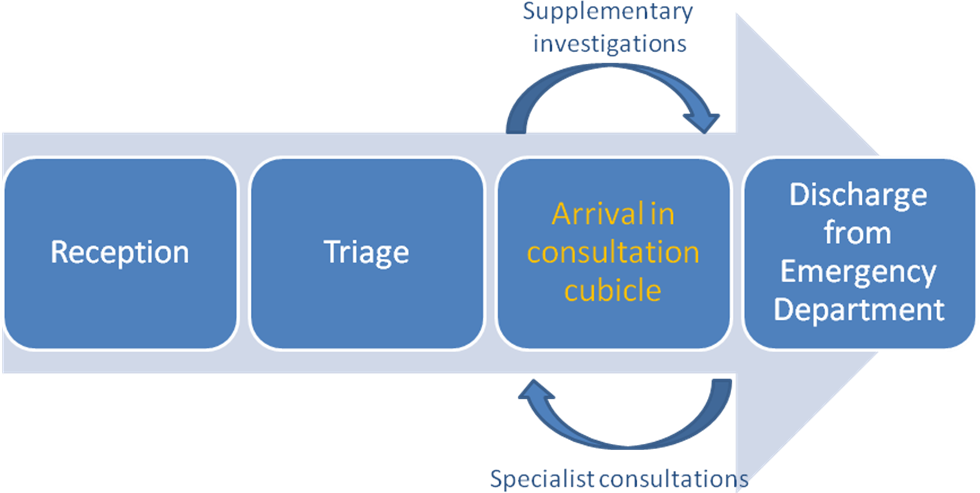


**7.2 Study plan**

**7.2.1. Training of the study investigator and health care personnel**

The study investigator will be trained to perform rapid HIV testing (anti-HIV antibodies) using the INSTI™ test which requires finger prick capillary blood. The health care personnel (ED doctors) will attend information sessions about the FOPH HIV testing recommendations and the study protocol, to be organised during a time scheduled for weekly departmental meetings.

**7.2.2. Method** (Figure 1)

The study investigator will select eligible ED patients based on their medical notes. After checking that no exclusion criteria are present (Annex 1), the patients will be randomised 1:1 between the two study arms. The randomisation will be performed in groups of 4-6 patients, grouped together randomly. The patients will be attributed to one or other study arm using the website [www.randomization.com](http://www.randomization.com/). The study arm will be written on a piece of paper inside an opaque envelope. The envelopes will be numbered consecutively and opened by the study investigator in numerical order at the time of randomisation. To avoid bias induced by the fact that the two testing approaches are detailed before completing any patient questionnaire, the step of providing information regarding the study and obtaining written consent will be performed once the questionnaire is completed or once information is presented by the electronic tablet and the offer of HIV testing has been made, depending on the study arm (Figure 1 and Annex 2). The study investigator will approach the patient and invite him or her to participate in a study on HIV screening in the ED. This introduction will be standardised and read from a prepared information sheet (Annex 8). For patients declining to participate in the study, only basic demographic data will be recorded (age, sex) without any patient identifier. This will order to allow comparison to be made between participants and patients eligible but not participating to quantify/exclude selection bias. For patients declining to participate after completing the study questionnaire, the responses and the decision regarding HIV testing will not be included in the final study analysis. Only basic demographic data (age, sex) without patient identifiers will be recorded.

Two groups of patients will therefore be included:

1. Patients receiving an electronic questionnaire based on the FOPH HIV testing criteria. After completing the questionnaire, a rapid test will be offered if it is recommended. It should be emphasized that the study investigator will have access only to the final recommendation on the tablet and not to the patients’ questionnaire responses.
2. Patients receiving the offer of non-targeted HIV screening via the electronic tablet after a short information on the relevance of this approach (Annex 3).

If the HIV test is accepted, an additional consent form must be signed (Annex 4), and the test will be performed by the study investigator during the ED consultation. Patients in group 1 (above) who report no HIV risk factors as listed in the questionnaire (Annex 5), will nonetheless be offered testing via the electronic tablet, offering them the possibility to benefit from their consultation by having a free test. Patients in group 2 who decline non-targeted screening will be invited automatically to complete the questionnaire received by group 1. If the questionnaire then ends with a recommendation for HIV testing related to their risk profile, they can then be tested.

After the electronic questionnaire, all participating patients will receive a paper questionnaire covering their socio-economic status, sexual orientation and whether or not they have a general practitioner (Annex 6). These data, together with demographic data obtained from the central hospital database, will enable analysis of patient characteristics of those accepting testing and of those declining. The patients will also be invited to give their opinion regarding the ease of use of the electronic tablet and the opportunity to be offered screening in the ED (Annex 6). The study investigator will remain nearby the ED cubicle to be available to offer assistance to participating patients, in using the electronic tablet or to explain questions in the questionnaire.

The study investigator will use a final questionnaire on the electronic tablet to record whether rapid HIV testing was accepted or declined and the test result when testing is performed. It will also be possible to record reasons for declining testing when applicable (Annex 7).

If the HIV rapid test is *reactive* (positive or indeterminate), the study investigator will inform the treating ED doctor who will in turn inform the patient of the result and explain that it is necessary to confirm the test result with a venous blood sample. The treating ED doctor will request the confirmatory test and immediately alert the duty HIV specialist at LUH. If the test is confirmed positive, the ED doctor will immediately organize an HIV specialist referral and outpatient follow-up in the Infectious Diseases Service.

For patients declining rapid HIV testing, the interview will be completed with the offer of information leaflets on the prevention of risk behaviour as well as places where HIV testing is offered in the region.

**Figure 1.** Schematic diagram representing the study protocol

**
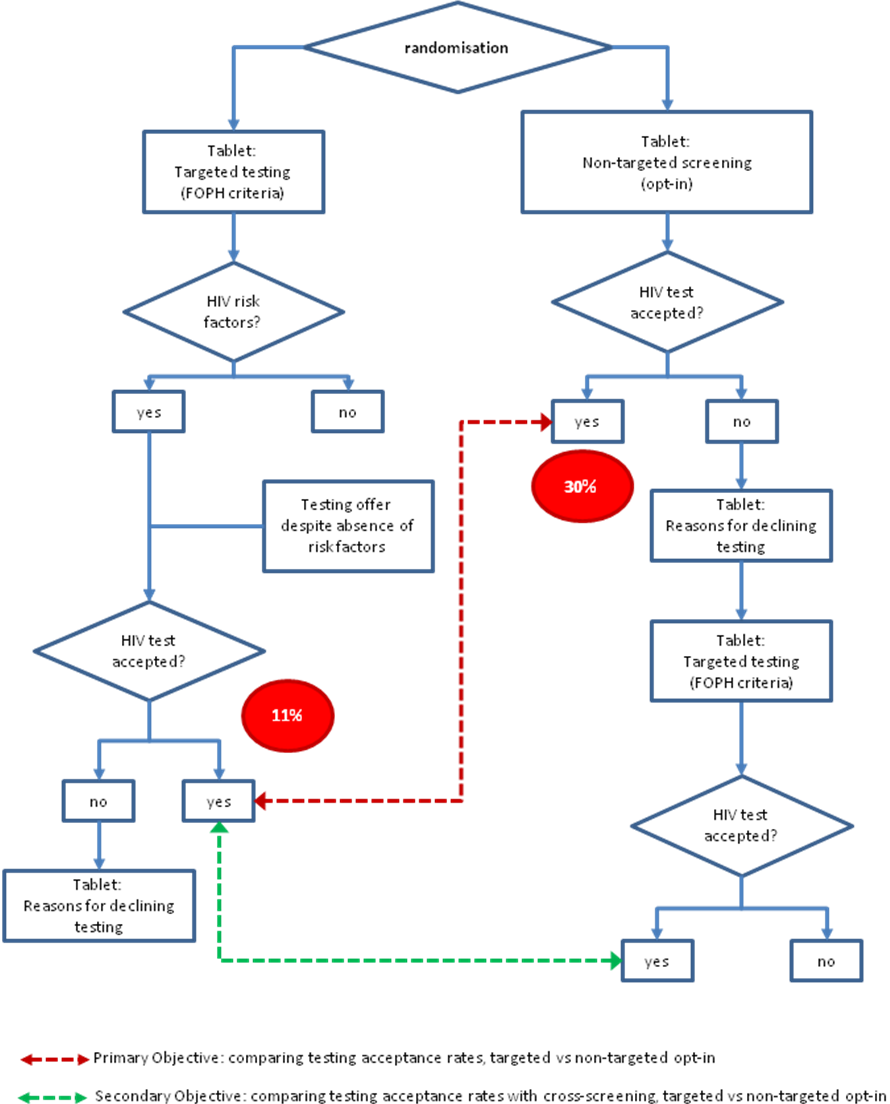
**

**7.2.3. Electronic questionnaire**

The questionnaire will be created using the website SurveyMonkey, a site often used at LUH for surveys. To guarantee anonymity and confidentiality of potentially sensitive data in a non-hospital database, no patient identifier will be recorded on this site. The online questionnaire will carry a unique study identifier for each participating patient. The study investigator will use this identifier on the paper questionnaire. The study investigator will then analyse the questionnaire responses using a database which will combine the data from the SurveyMonkey website, the central hospital database and the paper questionnaires, from which all identifiers (patient hospital number, hospital visit number, patient name) will be removed by the study supervisors.

- 1. **Parameters studied**

1. Patient demographic characteristics (from the central hospital database and from paper questionnaires, Annexes 1 and 5):
   - Age, sex, nationality, civil status, education level
2. Patient medical characteristics (from the central hospital database and from a paper questionnaire):

- Reason for presenting
- Diagnosis at the end of the ED visit
- Treatment proposed
- General practitioner (yes/no)

1. HIV screening (electronic tablet)

- Offered
- Accepted
- Performed
- Result (if performed)

1. HIV risk factors not detailed in the FOPH HIV testing recommendations:
   - Sexual orientation (paper questionnaire, Annex 5)
2. Reason(s) for declining testing if test not performed (questionnaire added to the electronic questionnaire as appropriate):

- Does not consider self at risk of HIV infection
- Recent negative HIV test and no risk behaviour since
- No time to perform test during ED visit
- Prefer to be tested by general practitioner
- Prefer to be tested at an anonymous testing centre
- Fear of the result
- Fear that family will hear of the result
- Fear that employer will hear of the result
- Other: (free text)

**7.4 Statistical analysis**

With the hypothesis that the offer of non-targeted opt-in HIV screening will increase the HIV testing rate by ~19% compared to a questionnaire on patient risk factors, 80 participants per group (non-targeted opt-in screening and targeted testing based on FOPH criteria) will be required to enable a power of 80% to demonstrate this difference, with a risk of type I error fixed at 5%.

**8. Medical follow-up**

Not applicable

**9. Role of nursing staff**

Not applicable

**10. Treatment**

Not applicable

**11. Evaluation of risk**

The offer of a rapid test (INSTI™) in this study is not sufficient to make the diagnosis of HIV infection. The rapid test must be coupled with a standard laboratory HIV test. The probability of a false positive rapid test is extremely low: 1/9072, accordingly to a multicentric French study [32]. The study participants will be informed of this risk in the information form which constitutes part of the consent form to be signed prior to rapid HIV testing.

In the case of potential at-risk exposure during the preceding 12 weeks, the study investigator will explain that a negative rapid test does not exclude acute HIV infection and that HIV testing must be repeated 12 weeks after the most recent exposure.

In the case of a *reactive* test (positive or indeterminate), the study investigator will inform the treating ED doctor who will contact the on-call HIV specialist at LUH and then send a venous blood sample for urgent HIV testing in the Immunology and Allergy laboratory. A definitive result will be obtained within 2 hours. It should be emphasized that the probability of a reactive test in the context of this study is very low: given that HIV seroprevalence in Switzerland is 0.4% and that around a third of positive individuals are unaware of their status, the probability of a positive test is around 0.2% in our group of 160 patients.

If the confirmatory test is negative, the treating ED doctor will give the result to the patient directly. If the confirmatory test is positive, the result will be given by the ED doctor who will be accompanied by a senior member of the LUH HIV team.

Conducting the study between 08:00H and 20:00H ensures that an HIV specialist will be present to engage the patient in care in the event of a positive test. As soon as the diagnosis is confirmed, antiretroviral therapy can be initiated as soon as possible, optimising the patient’s prognosis and minimising the risk of transmission to sexual partners.

There is currently a study in progress at LUH entitled, *Attitudes to and acceptance of HIV testing in the ED: a survey among doctor-patient pairs*, being conducted by two medical students in their second Masters year. The study is using rapid HIV testing offered by the study investigators (medical students) (Protocol N°95/14). The data collection phase has been completed without complication, with inclusion of 100 patients from LUH ED.

**12. Insurance cover**

In the event of injury caused to the study participants, LUH will respond in accordance with the institutional legal framework.

**13. Information and consent forms**

Attached as appendices (Annexes 1-8)

**14. Treatment of personal data and biological samples**

All patient data will be treated by the study investigator (Cléo Gillet) confidentially and anonymously. The data will be introduced anonymously into an Excel database. The database will be stripped of all patient identifiers, making it impossible to trace information back to the source patient. The electronic questionnaire using the website *SurveyMonkey* will have no questions which may reveal any patient’s identity.

**15. Funding**

The costs of this study will be met by the Faculty of Biology and Medicine, University of Lausanne, which provides some support for Masters projects and research funds from either Dr Cavassini or LUH ED. Costs arising in the event of a reactive HIV rapid test will be met by the patient’s health insurance.

**16. Participation of non-LUH health care personnel**

Not applicable

**17. Information for medical and paramedical health care personnel**

Information relating to the study will be provided to ED nurses and care assistants during their monthly team meetings in accordance with education practice in this ED.

**18. Cost**

HIV testing performed according to PICT is billed to the patient’s health insurance. Rapid HIV tests performed by the study investigator (Masters student) will not be billed to the patient. However, confirmatory tests will be billed to the patient’s health insurance provider.

CHF 9.50 per rapid HIV test: CHF 1,520- (maximum, with a testing rate at 100% of participants)

KD salary at 10% over 8 months:  CHF 8000-

Electronic tablet: CHF 300

*SurveyMonkey* website: CHF 450

**References**
