# Supplementary material for: Targeted versus non-targeted HIV testing offered via electronic questionnaire in a Swiss emergency department: A randomized controlled study
Source: PLoS One. 2018 Mar 7;13(3):e0190767. doi: 10.1371/journal.pone.0190767 (PMC5841645; doi:10.1371/journal.pone.0190767)
Supplement: S3 Text — (DOC) [file pone.0190767.s004.doc]

**Annex 1. Study questionnaires**

**Study questionnaire, Targeted group**

Thank you for agreeing to answer our questionnaire, your participation is very valuable to us. The answers you give to the following questions will be confidential and the investigator will not have access to them. Your answers will be anonymized and analyzed at a later stage. If you have any questions, do not hesitate to ask the study investigator for help.

1) How would you describe yourself, what is your sexual identity?

- Woman
- Man
- Transgender
- I do not wish to answer

2) What is the sexual identity of your partner(s)?

- Woman
- Man
- Transgender
- I do not wish to answer

3) Have you previously been tested for HIV?

- Yes
- No
- I do not wish to answer

4) If yes, when was the last time you got tested?

- 0-3 months ago
- 4-6 months ago
- 7-12 months ago
- 1-2 years ago
- 2-5 years ago
- 5-10 years ago
- >10 years ago

5) Which statement would you say applies to you

- I have had unprotected sexual intercourse with a man/men who has/have sexual intercourse with other men
- I have had unprotected sexual intercourse with one or more partners who have a sexually transmitted infection
- I have had unprotected sexual intercourse with one or more partners known to be HIV positive
- I have had sexual intercourse with one or more partners who inject drugs
- I have had sexual intercourse with one or more sex workers
- I have had sexual intercourse with one or more partners from sub-Saharan Africa, the Caribbean, Eastern Europe or South-East Asia, or while traveling in those regions
- I have injected drugs myself
- I already been for HIV and have had unprotected sexual intercourse since the last test.
- None of the above applies to me
- I do not wish to answer

1) Thank you for your answers on HIV risk behaviour. According to your answers, you have risk factors for HIV infection. According to official recommendations, you should be screened for HIV. The investigator can test you right now with a rapid test. It is free of charge and the result will be available in 3 to 6 minutes.

Do you wish to get tested for HIV with a rapid HIV test?

2) According to the answers you have given, you do not have any risk factors for HIV infection.

Do you wish to take the opportunity to get tested anyway? The test uses a drop of blood taken from the tip of the finger and gives a result in 3 to 6 minutes. It is free of charge.

3) The answers you have given in this questionnaire do not allow us to evaluate whether or not you are at risk of HIV infection.

Do you wish to take the opportunity to get tested anyway? The investigator could test you right now and it is free of charge.

->YES: You have agreed to get screened for HIV. You may now call the investigator who will come to test you.

->NO: You have declined an HIV test. Please allow us to take one more minute of your time in order to understand the reasons of your refusal.

What is the main reason for which you did not which to get tested?

- I do not believe I am at risk for HIV
- I am afraid of the possible result
- I don’t have time to take the test in the Emergency Department
- I recently got tested and the result was negative
- I am afraid my family could discover the result
- I am afraid my employer could discover the result
- I would rather get tested by my family physician
- I do not wish to answer
- Other

Are there other reasons why you do not wish to get tested?

- I do not believe I am at risk for HIV
- I am afraid of the possible result
- I don’t have time to take the test in the Emergency Department
- I recently got tested and the result was negative
- I am afraid my family could discover the result
- I am afraid my employer could discover the result
- I would rather get tested by my family physician
- I do not wish to answer
- Others

**Study questionnaire, non-targeted group**

Thank you for agreeing to help us in our study. Here is some information about HIV screening.

The percentage of the Swiss population infected with the virus of HIV, whch may lead to AIDS, is 0.4%.

This rate is low and yet high enough for a non-targeted screening to be justified according to HIV testing recommendations in other industrialised countries.

Non-targeted screening means an HIV test is offered to every individual seeking medical care, even if the reason for consulting has nothing to do with HIV (ankle sprain, headache, abdominal pain, etc).

There are multiple advantages to non-targeted screening:

- It allows early detection of HIV infections among people who are unaware they are infected. One in three patients in Switzerland is still diagnosed at a late stage of infection.
- AIDS is a disease that can be effectively treated in the 21^st^ century. A person infected with HIV and receiving treatment has a near-normal life expectancy.
- Early diagnosis enables early treatment, thus avoiding the possible consequences of the infection: neurological damage (memory), cardiovascular problems (ischaemic heart disease which can cause angina and heart attacks), as well as kidney and liver diseases.
- Being aware of an HIV diagnosis makes it possible to avoid infecting other people.
- Finally, the identification of all patients with HIV infection and starting treatment is an approach which will hopefully lead to eradication of the disease in the future.

The disadvantages of non-targeted HIV screening by rapid testing are:

- Needing to prick the fingertip with a small needle (once)
- Fear associated with the disease and of a possible positive result. However, the rapid test used in the study gives a result in 3 to 6 minutes so the person can be quickly reassured.
- The very low possibility (<0.1%) of a falsely reactive test, which means the test mistakenly indicates an HIV infection. Every reactive test is checked by a more precise laboratory test, which takes 2-3 hours to give a result.
- Finally, the rapid test does not detect infections that occurred less than 3 months ago. If you have potentially been exposed to HIV (unprotected sexual intercourse, drug injection) in the last three months, a laboratory test has to take place, or you can repeat the rapid test three month after the exposure.

Having read this information, would you like to get tested for HIV with a rapid test right now?
