# Supplementary figures and images for: Targeted versus non-targeted HIV testing offered via electronic questionnaire in a Swiss emergency department: A randomized controlled study
Source: PLoS One. 2018 Mar 7;13(3):e0190767. doi: 10.1371/journal.pone.0190767 (PMC5841645; doi:10.1371/journal.pone.0190767)

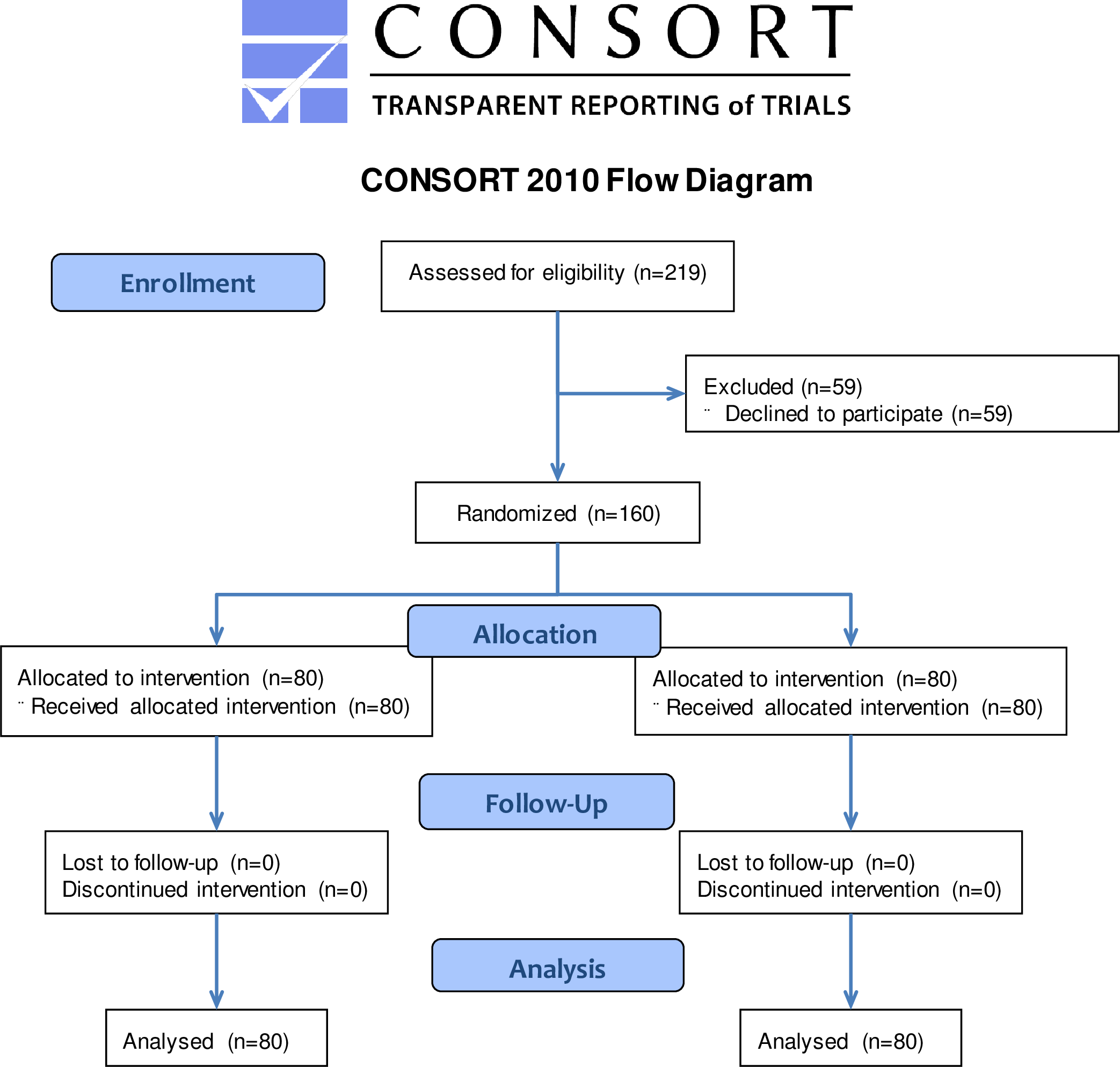

Supplement: S1 Fig — (TIF) [file pone.0190767.s005.tif]
